# Integrated UPLC-ESI-MS/MS, network pharmacology, and transcriptomics to reveal the material basis and mechanism of Schisandra chinensis Fruit Mixture against diabetic nephropathy

**DOI:** 10.3389/fimmu.2024.1526465

**Published:** 2025-02-19

**Authors:** Yuan-Yuan Deng, Xin-Yu Ma, Peng-Fei He, Zheng Luo, Ni Tian, Shao-Ning Dong, Sai Zhang, Jian Pan, Peng-Wei Miao, Xiang-Jun Liu, Cui Chen, Peng-Yu Zhu, Bo Pang, Jing Wang, Li-Yang Zheng, Xin-Kun Zhang, Min-Ying Zhang, Mian-Zhi Zhang

**Affiliations:** ^1^ Graduate School, Tianjin Medical University, Tianjin, China; ^2^ Department of Nephrology, Tianjin Academy of Traditional Chinese Medicine, Tianjin, China; ^3^ Second Clinical Medical College, Beijing University of Chinese Medicine, Beijing, China; ^4^ Department of Nephrology, Dongfeng Hospital, Beijing University of Chinese Medicine, Beijing, China; ^5^ College of Basic Medicine, Chengdu University of Traditional Chinese Medicine, Chengdu, China; ^6^ School of Clinical Medicine, Tianjin University of Traditional Chinese Medicine, Tianjin, China; ^7^ School of Medicine, Nankai University, Tianjin, China

**Keywords:** network pharmacology, diabetic nephropathy, Schisandra chinensis Fruit Mixture, JAK2/ATAT3 signalling pathway, inflammatory response, apoptosis

## Abstract

**Backgrounds:**

It has been regarded as an essential treatment option for diabetic nephropathy (DN) in Traditional Chinese medicine. Previous studies have demonstrated the anti-DN efficacy of Schisandra chinensis Fruit Mixture (SM); however, a comprehensive chemical fingerprint is still uncertain, and its mechanism of action, especially the potential therapeutic targets of anti-DN, needs to be further elucidated.

**Objective:**

Potential mechanisms of SM action on DN were explored through network pharmacology and experimental validation.

**Methods:**

The chemical composition of SM was analyzed using UPLC-ESI-MS/MS technology. Active bioactive components and potential targets of SM were identified using TCMSP, SwissDrugDesign, and SymMap platforms. Differentially expressed genes were determined using microarray gene data from the GSE30528 dataset. Related genes for DN were obtained from online databases, which include GeneCards, OMIM and DisGeNET. PPI networks and compound-target-pathway networks were constructed using Cytoscape. Functional annotation was performed using R software for GO enrichment and KEGG pathway analysis. The DN model was built for experimental validation using a high-sugar and high-fat diet combined with STZ induction. Hub targets and critical signaling pathways were detected using qPCR, Western blotting and immunofluorescence.

**Results:**

Utilizing the UPLC-ESI-MS/MS coupling technique, a comprehensive analysis identified 1281 chemical components of SM’s ethanol extract, with 349 of these components recognized as potential bioactive compounds through network pharmacology. Through this analysis, 126 shared targets and 15 HUB targets were pinpointed. Of these, JAK2 is regarded as the most critical gene. Enrichment analysis revealed that SM primarily operates within the PI3K/AKT signaling pathway. *In vivo* experiments confirmed that SM improved pathological injury and renal function in rats with DN while improving mitochondrial morphology and function and modulating the expression of proteins linked to apoptosis (cleaved-caspase-3, Bax, and Bcl-2) and pro-inflammatory factors (IL-6 and TNF-α). Mechanistically, SM alleviates DN primarily by suppressing the PI3K/AKT/mTOR and JAK2/STAT3 signaling pathways to fulfill the energy needs of renal tissues. Furthermore, molecular docking analysis provided direct validation of these findings.

**Conclusion:**

The findings of this study offer initial indications of the active component and robust anti-inflammatory and anti-apoptotic characteristics of SM in the mitigation of DN, along with its capacity to safeguard the integrity and functionality of mitochondria. This research unequivocally validates the favorable anti-DN effects of SM, indicating its potential as a viable pharmaceutical agent for the management of DN.

## Graphical Abstract

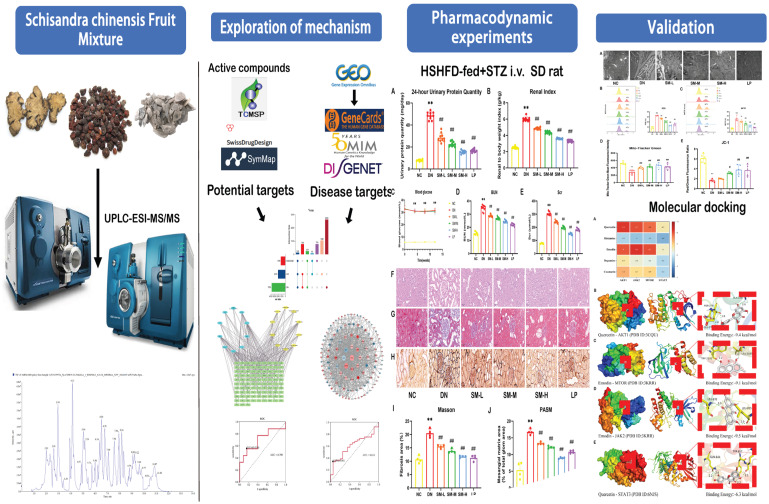

## 1 Introduction

Diabetic nephropathy (DN) is a significant microvascular complication arising from diabetes mellitus, which has emerged as a prevalent chronic metabolic disease globally, exhibiting a protracted clinical course and a substantial complication rate (1). Apoptosis, oxidative stress, and inflammatory responses have been implicated in the etiology of DN, ultimately leading to the deterioration of renal function (2). In clinical practice, DN is typically managed with a variety of drugs, including angiotensin-converting enzyme inhibitors (ACEi), angiotensin receptor blockers (ARBs), non-steroidal mineralocorticoid receptor antagonists (NS-MRAs), fexofenadine, and sodium-glucose cotransporter-2 (SGLT2) inhibitors. These drugs collectively aim to control blood pressure, reduce proteinuria, and preserve renal function (3). Prolonged use of these medications may lead to adverse effects such as persistent dry cough, urinary tract infections, and hypersensitivity reactions, which can limit their effectiveness (4, 5). Consequently, there is a pressing necessity to identify novel drugs.

Traditional Chinese medicine (TCM), specifically the Schisandra chinensis Fruit Mixture (SM) formulated by Professor Zhang Daning, is increasingly recognized as a beneficial strategy for the treatment of DN due to its empirical formula based on the principles of tonifying the kidney and activating the blood of the kidney (6). SM is a novel herbal formula comprising a combination of 3 herbal medicines: *Schisandra chinensis* fructus (Wuweizi; containing the dried fruiting body of *Schisandra chinensis* (Turcz.) Baill.), *Ligusticum chuanxiong Hort.* (Chuanxiong; containing dried roots and rhizomes of Ligusticum wallichii Franch), Ostreae concha (Muli; containing shells of three species of oyster: Crassostrea gigas Thunberg (Ostrea gigas Thunberg), Crassostrea talien-whanensis Crosse and Crassostrea rivularis Gould). Recent research has shown that herbal formulas with properties of kidney tonification and blood activation can effectively mitigate fibrotic injury in mice with streptozotocin (STZ)-induced DN, thereby preserving renal function (7). Previous investigations conducted by our research group have also demonstrated the ability of SM and its bioactive compounds to attenuate renal fibrosis in DN (8, 9). While these findings suggest the therapeutic potential of SM in treating DN, further research is needed to elucidate the specific components and mechanisms underlying its efficacy.

Phytochemical analysis utilizing ultrahigh-performance liquid chromatography-electrospray ionization-tandem mass spectrometry (UPLC-ESI-MS/MS) has proven to be a valuable analytical technique for identifying the bioactive compounds present in herbal remedies (10, 11). In addition, with the development of high-throughput sequencing technology, transcriptome and microarray profiling have been widely used in a variety of diseases, including DNA (12). In addition, with the development of high-throughput sequencing technology, transcriptome and microarray profiling have been widely used in a variety of diseases, including to find biomarkers of disease progression, gain a deeper understanding of the pathogenesis of DN, and develop new strategies for individualized treatments. In recent years, the integration of network pharmacology and transcriptomics has emerged as a significant approach for elucidating the mechanisms underlying the therapeutic effects of natural products. This method facilitates the identification of molecular and pharmacological pathways, thereby enhancing our understanding of the complex interactions involved in multi-targeted herbal formulations (13, 14).

This study utilized a combination of UPLC-ESI-MS/MS-based widely targeted metabolomic methods, network pharmacology, and transcriptomics to identify and predict the potential active components and targets of SM for the treatment of DN. Additionally, a rat model of DN induced by a high-sugar, high-fat diet (HSHFD) combined with STZ was established to confirm the predictive analyses and elucidate the underlying mechanisms of SM in treating DN. The **Graphical Abstract** is shown here.

## 2 Materials and methods

### 2.1 Preparation of SM ethanol extract and UPLC-ESI-MS/MS analysis

The herbs used in this study were sourced from the Tianjin Chinese Medicine Decoction Piece Co., Ltd., which included *Schisandra chinensis* fructus (batch number G2011040-03), *Ligusticum chuanxiong Hort.* (batch number 9210800101), and Ostreae concha (batch number G2104019-01), and verified by Professor Mianzhi Zhang. The authenticated samples were stored at the Experimental Centre of Dongfang Hospital, Beijing University of Chinese Medicine. The composition of SM was determined based on the specifications outlined in **Supplementary Table 1**. The extraction process for the specific drugs is detailed as follows. Initially, the mixed herbs were subjected to extraction by soaking them 8 times the volume of 95% ethanol (v/w) for a duration of half an hour, followed by heating and refluxing for 3 hours. The resulting solution was then filtered, and the residue from the filtration process was subjected to re-extraction by heating and refluxing with six times the weight of the original herb in 95% ethanol for 2 cycles, each lasting 3 hours. Subsequently, the solutions obtained from the three extraction processes were combined and concentrated to a relative density of 1 g/ml before being stored at a temperature of 4°C.

The 2 ml sample was weighed and placed in a 50 ml centrifuge tube, which was extracted with 1200µl of 70% methanol. After 1 minute, the sample underwent centrifugation at 12000 r/min, maintained at 4°C, for 3 minutes. Subsequently, the supernatant was filtered through a microporous filter membrane, possessing a pore size of 0.22 μm, and was subsequently analyzed using UPLC-ESI-MS/MS.

### 2.2 Strategy of identification and characterization of compounds

The analytical conditions were as follows: UPLC column, Agilent SB-C18 (1.8 µm, 2.1 mm * 100 mm); the mobile phase consisted of solvent A, pure water with 0.1% formic acid, and solvent B, acetonitrile with 0.1% formic acid. Sample measurements were performed with a gradient program that employed the starting conditions of 95% A, 5% B. Within 9 min, a linear gradient to 5% A, 95% B was programmed, and a composition of 5% A, 95% B was kept for 1 min. Subsequently, a composition of 95% A and 5.0% B was adjusted within 1.1 min and kept for 2.9 min. The flow velocity was 0.35 mL per minute, and the column oven was set to 40°C. The injection volume was set to 2 μL. The effluent was alternatively connected to an ESI-triple quadrupole-linear iontrap (QTRAP)-MS. The ESI source operation parameters were as follows: source temperature 550°C; ion spray voltage (IS) of 5500 V (positive ion mode) and -4500 V (negative ion mode); ion source gas I (GSI), gas II (GSII), and curtain gas (CUR) were set at 50, 60, and 25 psi, respectively; the collision-activated dissociation (CAD) was high.

### 2.3 Target prediction of SM bioactive ingredients

Composition was performed on the screened substances using TCMSP (https://www.tcmsp-e.com//), SwissDrugDesign and SymMap (http://www.symmap.org/) databases, respectively, with the following screening criteria: the GI absorption has been set to “HIGH,” and two or more compounds can be considered active ingredients if they pass all five predictive properties (Lipinski, Ghose, Veber, Egan and Muegge) by SwissADME program (http://swissadme.ch/index.php); exhibiting oral bioavailability (OB) of at ≥ 30% and drug-likeness (DL) of at ≥ 0.18 in the TSMSP database were deemed as the bioactive ingredients of SM. Then, we searched the TCMSP database, SymMap (http://www.symmap.org/), and SwissTargetPrediction database for predicted mapped targets. Next, the UniProt database (https://www.uniprot.org/) was applied by entering the target name and limiting the species to human. All retrieved targets were corrected to the gene symbol and the target corresponding to the compound was obtained.

### 2.4 Analysis of transcriptomics data and gene set enrichment analysis

The GSE30528 dataset was obtained from the GEO public database. It includes genes from the glomerular tissue of nine DN patients and thirteen normal controls on the GPL571 platform. To identify the corresponding genes, we utilized probe IDs within the Bioconductor package of the R software. In cases where a gene had multiple probes on the same chip, the average expression value of all those probes was considered representative of the gene’s expression level. To identify differentially expressed genes (DEGs), we utilized the Benjamini-Hochberg corrected two-tailed t-test to control for false discovery rates and detect genes with significant expression changes. DEGs were further filtered (*p* < 0.05 and |log2 fold change (FC)| > 1) using the Limma package (15) in R. Visualization of DEGs expression patterns was achieved through the generation of heatmaps and volcano plots using the ‘Pheatmap’ and ‘ggplot2’ packages in R (16), respectively. Analyzing and interpreting pathway-level changes between normals and DNs are done using gene set enrichment analysis (GSEA) (17). It was performed with the R package clusterProfiler (18), using the MSigDB GeneSet Database [H: hallmark gene sets (50)] as the reference genome and 10,000 alignments, with a significance threshold of 10. We visualized the results with the R package “ggplot2”.

### 2.5 Acquisition of disease-related targets

DN-associated genes were obtained from GeneCards (https://www.genecards.org) (screening threshold: relevance score greater than 0), OMIM (http://www.omim.org), and DisGeNET (https://www.disgenet.org) online databases using the keyword “diabetic nephropathy”.

### 2.6 Functional enrichment analysis of potential targets

GO and KEGG analyses were conducted using the R software package (org.Hs.eg.db, colorspace, stringi, DOSE, clusterProfile, ggplot2), which can be downloaded from the Bioconductor online site (https://www.bioconductor.org/) (19), to performed ID conversion, GO functional analysis, and KEGG pathway analysis. The screening criteria were set at a significance level of *p* < 0.05 to examine the biological processes (BP), molecular function (MF), and cellular components (CC) associated with the DEGs.

### 2.7 Construction of a PPI network and hub gene screening

The genes with overlapping regions were inputted into the STRING database (https://cn.string-db.org/) with a confidence score of 0.4, leading to the construction of a PPI network by removing unconnected nodes. The interactions among the various genes were retrieved, and the PPI network was refined utilizing Cytoscape software (v.3.8.2). The structure of the PPI network was assessed using the Network Analyzer plug-in, and an examination of node degree values was conducted to identify the central target gene network. JAK2 was identified as a HUB gene and subsequently verified in the GSE96804 dataset. To estimate the discriminatory capability of JAK2 in distinguishing DN patients from healthy controls, the ROC (20) was plotted utilizing the “pROC” package in R software.

### 2.8 Active ingredient-target-disease network diagram construction

The Cytoscape software was utilized to construct a ‘component-target-pathway’ network to investigate the anti-DN mechanism of active compounds in SM.

### 2.9 Molecular docking studies

It was determined whether there are any potential interactions between the target and key bioactive components through molecular docking (21). The crystal structures of the key target genes were retrieved from the PDB online database (22). Details of the critical proteins are shown in **Supplementary Table 1**. Subsequently, the 3D structural representations of the ligands were obtained from the PubChem database (23) (https://pubchem.ncbi.nlm.nih.gov/), which were comprised of Coumarin (PubChem CID: 323), the primary active component of SM, Dopamine (PubChem CID: 681), Emodin (PubChem CID: 3220), Histamine (PubChem CID: 774), and quercetin (PubChem CID: 5280343). Next, the small molecule structure was optimized using the MMFF94 force field of the OpenBabel toolbox (24) to finally obtain the optimal molecular structure for the lowest energy state. Proteins were hydrogenated using AutoDock Tools 1.5.6, and small molecules were hydrogenated and determined to be torsionally bonded and saved as pdbqt files. Use the Grid plate to set the molecular docking range parameters, and the docking range parameters for each protein are shown in **Supplementary Table 2**. Subsequently, Auto Dock Vina 1.2.0 software (25) was run to docking model to calculate the affinity between receptor and ligand. The results were visualized using PyMOL (v.2.3.0) software (26).

### 2.10 Experimental validation *in vivo*

#### 2.10.1 Experimental animals

Sixty-five male Sprague-Dawley (SD) rats of clean-grade quality, aged 6 weeks with a body mass of 200 ± 20 g, were procured from Beijing Viton Lihua Laboratory Animal Technology Co. Ltd (Laboratory Animal License No. SYXK (Beijing) 2019-0013). The SD rats were housed in a specific pathogen-free (SPF)-grade animal facility at the Experimental Animal Centre of Dongfang Hospital, University of Chinese Medicine, Beijing, China. The housing conditions included a temperature of 23 ± 2°C, humidity maintained within the 40% - 60% range, light and dark cycle of 12 hours, and ad libitum access to water and food. All procedures in this study followed internationally recognized principles for using and caring laboratory animals and were approved by the Animal Protection and Use Committee of the Oriental Hospital of Beijing University of Chinese Medicine ((approval number: DFYY202102R).

#### 2.10.2 Experimental method

Following a one-week acclimatization period, the rats were divided into the normal group (NC, n=10) and the model group (M, n=55). The M group was subjected to an HSHFD consisting of a 67% maintenance diet, 10% lard, 20% sucrose, 2.5% cholesterol, and 0.5% sodium cholate, while the NC group continued with a standard diet. After six weeks of unrestricted feeding, diabetes was induced in the M group through intraperitoneal injection of 35 mg/kg STZ (Sigma-Aldrich, S0130-1G) freshly dissolved in sodium citrate buffer (0.1 mmol/L, pH 4.5, Solarbio, C1013). In contrast, rats in group NC were injected with an equal volume of sodium citrate buffer intraperitoneally.

**Figure 1:**
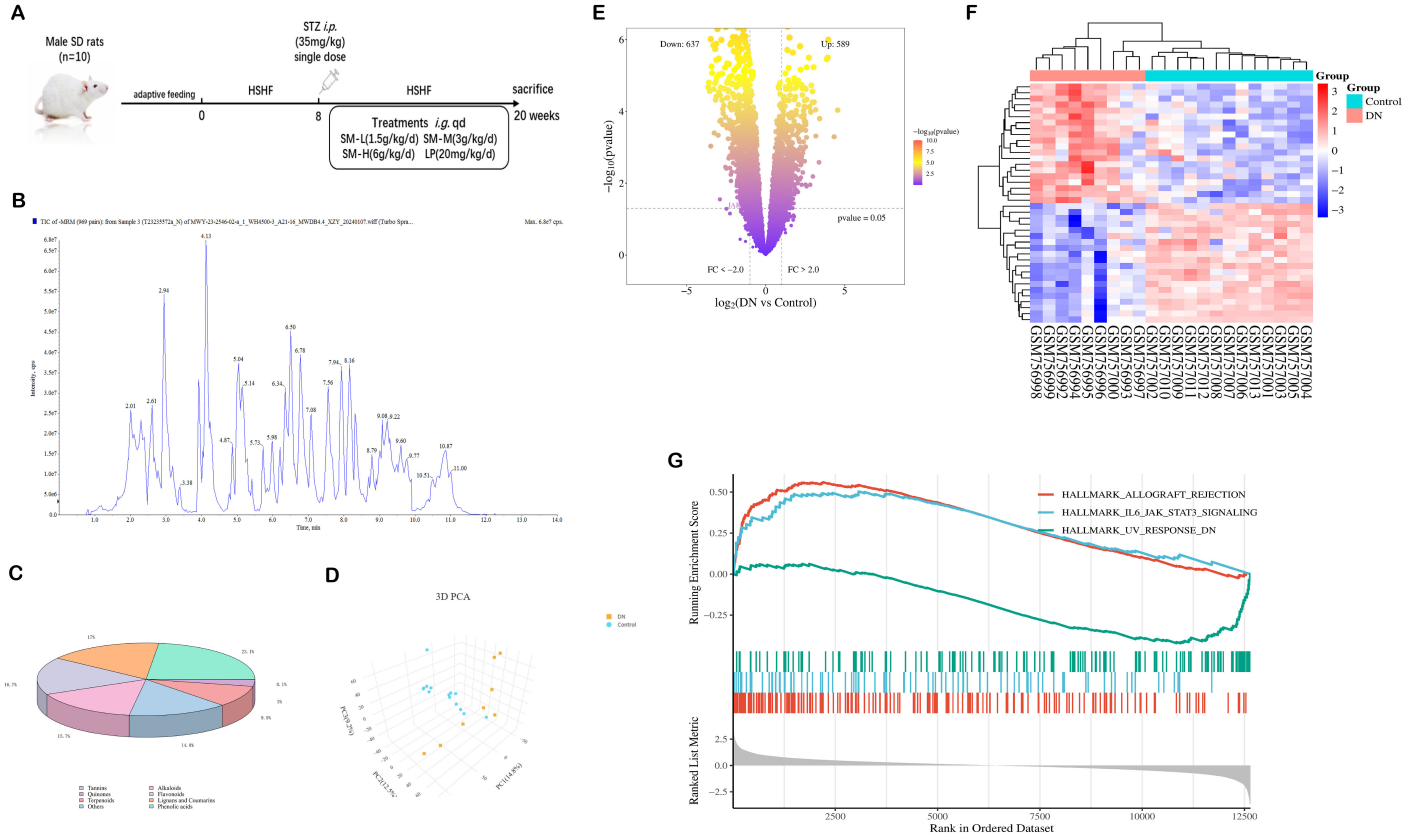
Identification of SM chemistry and acquisition of DN targets. **(A)** Experimental design. **(B)** The total ion current is based on a UPLC-ESI-MS/MS chromatogram of SM’s ethanol extract. **(C)** Categorized pie chart of SM components. **(D)** PCA diagram of GSE30528. **(E)** Volcano map of DEGs in GSE30528. **(F)** Heat map of DEGs in GSE30528. **(G)** GSEA Analysis in GSE30528.

Random blood glucose levels were assessed using a Roche blood glucose meter after 72 hours. Rats were deemed to have effectively simulated DN if they exhibited a random blood glucose concentration of ≥ 16.7 mmoL/L (300 mg/dL) for three consecutive days and a 24-hour urine protein quantification of ≥ 30 mg/24h (27). Five rats died in the process and were excluded, and rats with successful modelling (n = 50) were used for further studies. Next, M group was randomly divided into 5 groups: (1) DN (n=10), (2) SM-L (1.5 g/kg/d, n = 10), (3) SM-M (3 g/kg/d, n = 10, (4) SM-H (6 g/kg/d, n = 10), (5) Losartan Potassium (LP) (20 mg/kg/d, n = 10), which was administered continuously for 12 weeks. Gastric gavage gave equal volumes of double-distilled water in the DN and NC groups. As illustrated in **Figure 1A**, the experimental methodology involved gavage administration at specified time intervals, with regular monitoring of body weight and random blood glucose levels every 4 weeks. Upon reaching week 20, all rats were humanely euthanized, and serum and tissue samples were obtained. A portion of the kidney was preserved in 4% paraformaldehyde for histological examination, while the remaining portion was promptly frozen for subsequent molecular analysis. The experimental design is shown in **Figure 1A**.

#### 2.10.3 Measurement of 24-hour quantitative urine protein

The 24-hour urine of rats in each group was collected in metabolic cages and mixed well. The supernatant was aspirated after centrifugation (3500 rpm × 20 min at four °C), and the quantification of 24-h urinary protein was determined by a urinary protein kit (Nanjing JianCheng, C035-2-1). The 24-hour urinary protein was calculated and detected every 4 W.

#### 2.10.4 Measurement of biochemical indicators

Blood samples were obtained from the abdominal aorta of all rats and subsequently centrifuged (3500 rpm × 15 min at 4°C) after a 3-hour incubation at room temperature to isolate serum samples to assess baseline renal function. Serum urea nitrogen (BUN) levels were determined using a blood urea nitrogen kit (NanJing JianCheng, C013-2-1) and serum creatinine (Scr) was measured using a blood creatinine kit (NanJing JianCheng, C011-2-1) as per the specifications provided by the reagent vendors.

#### 2.10.5 Measurement of the kidney to body weight ratio

After execution, both kidneys were removed and weighed, as described previously, and the ratio of total kidney weight (mg)/rat body weight (g) was calculated. A kidney-to-body weight ratio can also indirectly reflect the degree of renal fibrosis as a quantitative indicator of kidney hypertrophy.

#### 2.10.6 Histological analysis

The kidneys were harvested and immersed in a 4% paraformaldehyde solution for 48 hours, followed by dehydration, paraffin embedding, and sectioning at a thickness of approximately 3 μm. Subsequently, staining techniques, including hematoxylin and eosin (H&E), periodic acid silver methylamine (PASM), and Masson’s trichrome (Masson), were conducted.

#### 2.10.7 Transmission electron microscopy observation of mitochondria

Fresh kidney tissue samples measuring 1 mm^3^ were fixed in 2.5% glutaraldehyde solution prepared in 0.1 mol/L phosphate buffer (pH = 7.4) for 2 hours at 4°C using a pre-chilled blade. Subsequently, the samples underwent three washes with 50 ml of phosphate buffer (pH = 7.4, 0.1 mol/L). The kidney tissues were extracted and reintroduced into 1% osmium tetroxide, fixed at 4°C for 2 hours, followed by two washes with phosphate buffer. Dehydration was carried out in a series of graded ethanol concentrations. Next, immersion, embedding, and polymerization were performed. Finally, the ultrathin sections (50 nm thick) were observed and photographed using a TEM after double staining with dioxiranyl acetate and lead citrate for 20 min.

#### 2.10.8 Terminal deoxynucleotidyl transferase-mediated dUTP nick end labeling staining

Apoptosis was detected in kidney tissues with the One-step TUNEL *In Situ* Apoptosis Kit (Elabscience, E-CK-A320) following the manufacturer’s instructions. Then, the nuclei were stained with DAPI. Finally, images were acquired with a fluorescence microscope (Nikon, Japan).

#### 2.10.9 Measurement of mitochondrial content (MitoTracker Green), the mitochondrial membrane potential, mitochondrial permeability transition pore and reactive oxygen species

Fresh kidney tissues were obtained, and single-cell suspensions of *in vivo* kidney tissues were prepared. The cells were then incubated in Calcein AM staining solution and DCFH-DA for 30 minutes at 37°C respectively, shielded from light, following the established protocols of the MPTP assay kit (Beyotime, C2009S) and ROS assay kit (Beyotime, S0033S). Subsequently, after washing, centrifugation, and resuspension, the average fluorescence intensity was quantified using flow cytometry to evaluate the extent of MPTP and ROS opening.

Mitochondrial activity was measured utilizing the MitoTracker Green kit (Yeasen, 40742ES50).

The staining working solution was prepared according to the Mito Tracker^®^ Green FM kit, and after washing with PBS, the treated cells were stained with Mitotracker Green FM. Fluorescence intensity was scanned and analyzed at excitation/emission wavelengths (490/523 nm) using a fluorescent enzyme marker (Molecular Devices, USA).

According to the literature, Mitochondria were extracted and purified from kidney tissue using the Mitochondrial Extract Kit (Servicebio, SM0020). Then, changes in MMP were detected using the MMP assay kit (Beyotime, C2003S) containing JC-1. Purified mitochondria were stained with JC-1 staining solution at 37°C for 20 min. Fluorescence intensity was scanned and analyzed using a fluorescence enzyme marker (Molecular Devices, USA). JC-1 monomer expression was detected at an excitation wavelength of 490 nm and an emission wavelength of 530 nm. Red fluorescent JC-1 aggregates are observed in hyperpolarized membranes, while the green fluorescent monomeric form indicates membrane depolarization. Thus, MMP was determined by analyzing the reduced red/green fluorescence intensity ratio, with higher values indicating a more structurally intact mitochondrial membrane.

#### 2.10.10 Reverse transcription-quantitative polymerase chain reaction

Total RNA was extracted using Trizol reagent following the manufacturer’s instructions, and RNA integrity was evaluated by spectrophotometry at 260 nm. Subsequently, RNA was reverse transcribed to cDNA utilizing a reverse transcription kit (ExonScript RT SuperMix with dsDNase) with reaction conditions set at 25°C for 10 min, 55°C for 15 min, and 85°C for 5 min. The cDNA product was used as a PCR template, and the reaction conditions for real-time PCR were as in **Supplementary Table 3**. The relative expression levels of target genes (Caspase-3, Bcl-2, and Bax m RNA) were determined using the 2-ΔΔCt method with β-actin as the internal reference. The amplification was carried out using a fluorescence quantitative PCR instrument. Primer sequences can be found in the **Supplementary Table 4**.

#### 2.10.11 Enzyme-linked immunosorbent assay

The levels of IL-6 (Lianke, EK306) and TNF-α (Lianke, EK382) in renal tissues were determined by ELISA kits according to the instructions of the reagent vendor.

#### 2.10.12 Western blot analysis

Remove the unnecessary punctuation and clarify the steps more formally: 100 mg of rat kidney tissue was removed from a -80°C freezer and added to RIPA tissue lysate. The mixture was homogenized on ice and allowed to stand for 10 minutes before being centrifuged at 12,000 rpm for 15 minutes at 4°C to collect the supernatant. The total protein content was determined using the BCA Protein Quantification Kit, and separation gels and stacked gels were prepared. The protein was transferred to a PVDF membrane via SDS-PAGE electrophoresis. The gel blocking was performed with 5% nonfat dry milk in Tris-buffered saline containing 0.1% TBST at room temperature for 1 h, followed by incubation with primary antibodies overnight at 4°C. Primary antibodies include AKT (1:1000, Proteintech, 60203-2-Ig), p-AKT (1:1000, Proteintech, 80455-1-RR), mTOR (1:1000, Proteintech, 28273-1-AP), p-mTOR (1:1000, Biodragon, BD-PP0176), JAK2(1:1000,Biodragon, BD-PT2426), p-JAK2 (1:1000, Biodragon, BD-PP1374), STAT3 (1:1000, Biodragon, BD-PT4443), p-STAT3 (1:1000, Biodragon, BD-PP1513), cleaved-Caspase-3 (1:1000, proteintech, 25128-1-AP), Bc1-2 (1:1000, Biodragon, BD-PT0470), Bax (1:1000, Biodragon, BD-PT0455) and β-actin (1:3000, Affinity, AF7018). Following three washes with TBST, the membrane was incubated with the appropriate secondary antibody for 1 hour at room temperature. Protein bands were visualized and quantified using ECL and Image J software.

#### 2.10.13 Immunofluorescence

Frozen renal tissues were sectioned (5 μm), fixed, and washed with PBS buffer before incubating with a 5% sealing serum at room temperature for 1 hour. The sealing solution was then removed, and the tissues were incubated overnight at 4°C with primary antibodies (anti-p-AKT, 1:100; anti-p-mTOR, 1:200; anti-p-JAK2, 1:50; p-STAT3, 1: 50). The following day, the sections were washed with PBS and incubated with corresponding secondary antibodies at room temperature for 1 hour in the dark. After the second antibody incubation, the tissue sections were washed with PBS and incubated with DAPI in the dark for 10 minutes. Next, the sections were sealed by adding an anti-fluorescent bursting agent and visualized by fluorescence microscopy.

### 2.11 Statistical analysis

Experimental data were collected with the mean expressed as ± standard deviation (SD). Each independent experiment was replicated a minimum of three times to determine the mean. Student’s t-test was utilized for comparisons between two groups, while one-way ANOVA and *post hoc* Tukey’s test were employed for comparisons among multiple groups. Wilcoxon paired-signs rank test was used for comparisons between groups with non-parametric data distributions. Statistical analyses and graphical representations were performed using GraphPad Prism 8.0. A significance level of *P* < 0.05 was considered statistically significant.

## 3 Results

### 3.1 Results of the identification of the components of the alcoholic extract of SM

#### 3.1.1 Identification and characterization of constituents of SM

The chemical composition of SM was thoroughly analyzed using UPLC-ESI-MS/MS, with the total ion chromatogram in negative ion mode depicted in **Figure 1B**. A total of 1281 chemical components in SM were identified based on the MS data and standard fragmentation pattern. These constituents were further categorized into 8 classes, as illustrated in **Figure 1C**.

### 3.2 Results of network pharmacological analyses

#### 3.2.1 Results of prediction and enrichment analysis of the active components of SM

The 1281 compounds were subsequently inputted into TCMSP, SwissDrugDesign, and SymMap databases for active ingredient screening and target prediction of SM. A total of 349 SM bioactive components (**Supplementary Table 5**) and 2025 corresponding targets were annotated.

#### 3.2.2 The results of prediction for biomarkers of DN

The results of principal component analysis (PCA) depicted in **Figure 1D** revealed significant distinctions between the populations of the DN and Control groups within the GSE30528 dataset, suggesting the credibility of the sample sources. Subsequently, a total of 1 226 DEGs were obtained (**Figure 1E**). As shown in **Figure 1F**, the results of the clustered heatmap indicated that the DEGs were able to clearly differentiate between DN tissues and normal tissues, and thus, the DEGs were representative of the whole sample. GSEA (**Figure 1G**) showed that the DEGs from the GSE30528 dataset were mainly involved in pathways related to inflammation, energy metabolism and immunity, including IL6/JAK-STAT3 signaling, inflammation response, oxidative phosphorylation, epithelial-mesenchymal transition, UA response DN, TGF - β signaling, etc.

#### 3.2.3 Prediction for DN-related target genes of GeneCards, OMIM, and DisGeNET

Three databases were consulted to collect 3760 DN-related target genes: GeneCards, OMIM, and DisGeNET.

#### 3.2.4 Construction of PPI network, identification and validation of HUB genes

**Figure 2:**
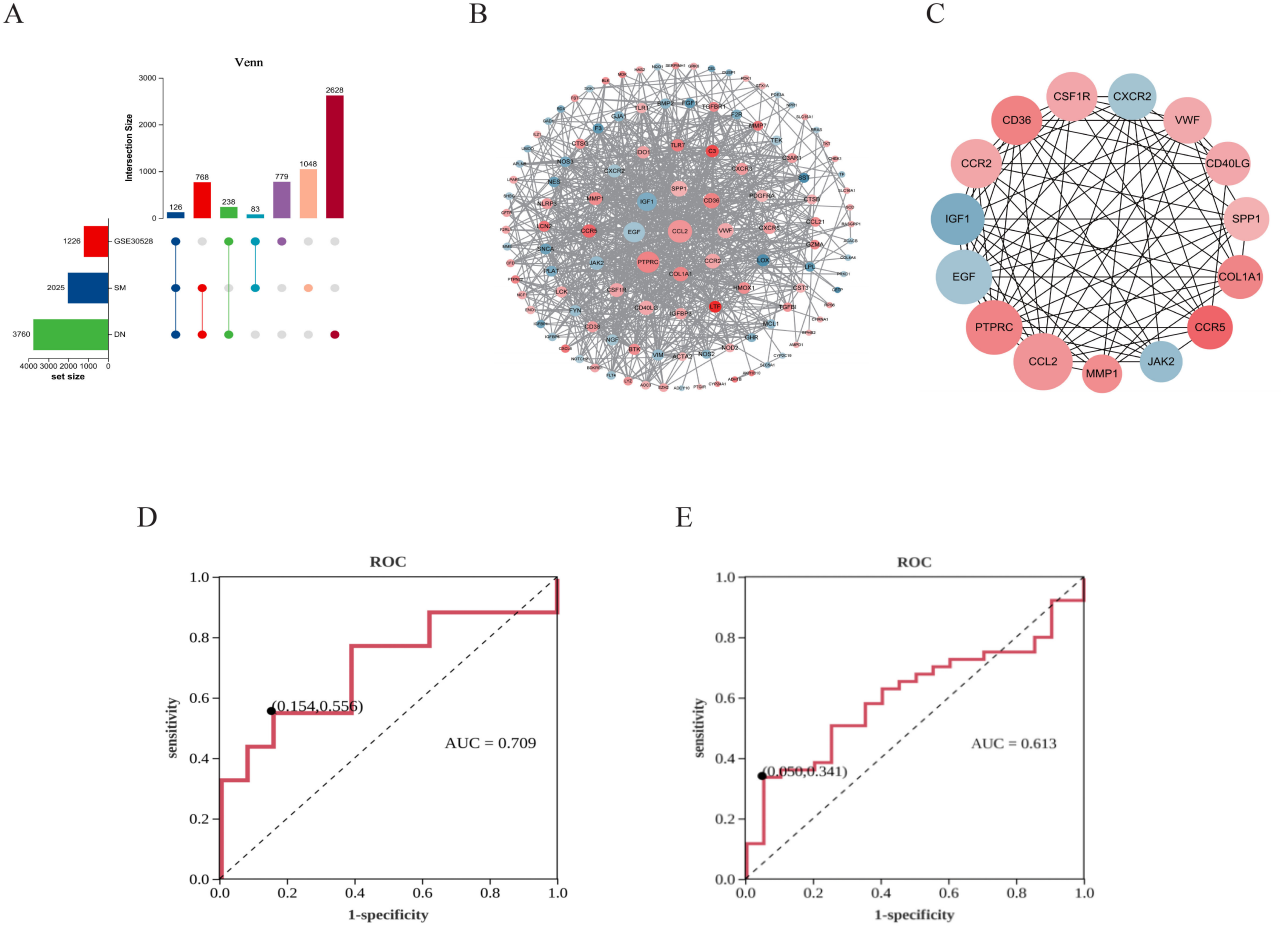
Identification results of critical targets for SM therapy DN. **(A)** UpSet diagram. **(B)** PPI network. **(C)** Network of HUB genes. **(D)** ROC analysis of JAK2 in GSE30528. **(E)** ROC analysis of JAK2 in GSE96804.

The UpSet plot revealed that a collective sum of 126 shared genes was identified from SM, DN, and GSE30528 datasets (**Figure 2A**). Subsequently, the significance of these potential targets was assessed through network topology analysis in PPI analysis. After removing isolated nodes, the network comprised 120 nodes and 769 edges. The significance of nodes within the network was assessed based on their degree. Furthermore, in the PPI network graph (**Figure 2B**), red nodes denote up-regulated genes, while blue nodes represent down-regulated genes. The intensity of the color is directly proportional to the absolute value of the log2 FC, and the size of the nodes is determined by their degree (higher-degree nodes are positioned closer to the network center and have larger areas). The top 15 protein targets with mean degree values greater than 24 included CCL2, PTPRC, EGF, IGF1, SPP1, CD36, VWF, COL1A1, CCR2, CD40LG, CSF1R, JAK2, CCR5, CXCR2, and MMP1, which were identified as HUB genes (**Figure 2C**). The ROC curve of JAK2 in GSE30528 exhibited an AUC of 70.9%, specificity of 84.6%, and sensitivity of 55.6% (**Figure 2D**). Additionally, a validation set (GSE96804) was utilized to enhance the accuracy and reliability of the results, confirming the diagnostic ability and expression level of JAK2. The AUC of JAK2 was determined to be 61.3% (**Figure 2E**), indicating its importance as a biomarker for DN with significant diagnostic potential. These results demonstrated that JAK2 is a crucial biomarker for DN, with significant diagnostic value for DN. Detailed information on the core targets is provided in **Supplementary Table 6**.

#### 3.2.5 Functional analysis of DN-related genes of SM

**Figure 3:**
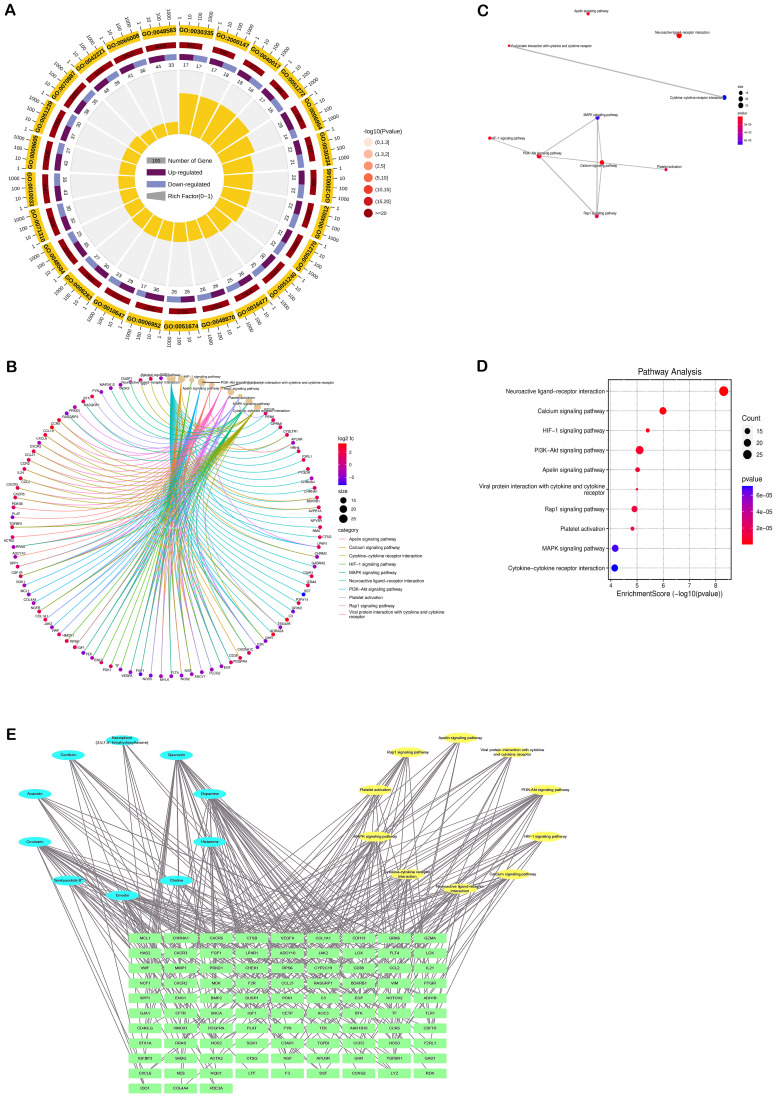
Results of enrichment analysis of the DN-related targets of SM. **(A)** Chord diagram of GO pathway. **(B-D)** KEGG analysis. **(E)** Network of component-target-pathway. The blue ovals represent the active ingredients of the SM, the yellow oval represents the pathway, and the green diamond represents the target.

To elucidate the potential functions of the overlapping genes, gene annotation and functional enrichment analyses were performed using R software (**Figures 3A–D**). GO enrichment analysis were mainly involved in treating 2 major aspects of DN injury, including inhibition of apoptosis and inhibition of inflammation. The KEGG pathways related to these targets (*p* < 0.05) included pathways in (**Figures 3B–D**): PI3K/AKT signaling pathway, Rap1 signaling pathway, MAPK signaling pathway, Calcium signaling pathway, HIF-1 signaling pathway, etc. These pathways are mostly associated with apoptosis, inflammatory response, and mitochondrial function.

#### 3.2.6 Component-target-pathway maps and key components

The ‘component-target-pathway’ network (**Figure 3E**) showed that Quercetin was the most active component of SM, followed by Dopamine, Coumarin, Emodin, and Histamine, with degree values of 511, 320, 320, 237, and 182, respectively.

### 3.3 Experimental verification

#### 3.3.1 Therapeutic effect of SM in STZ-induced DN rats

**Figure 4:**
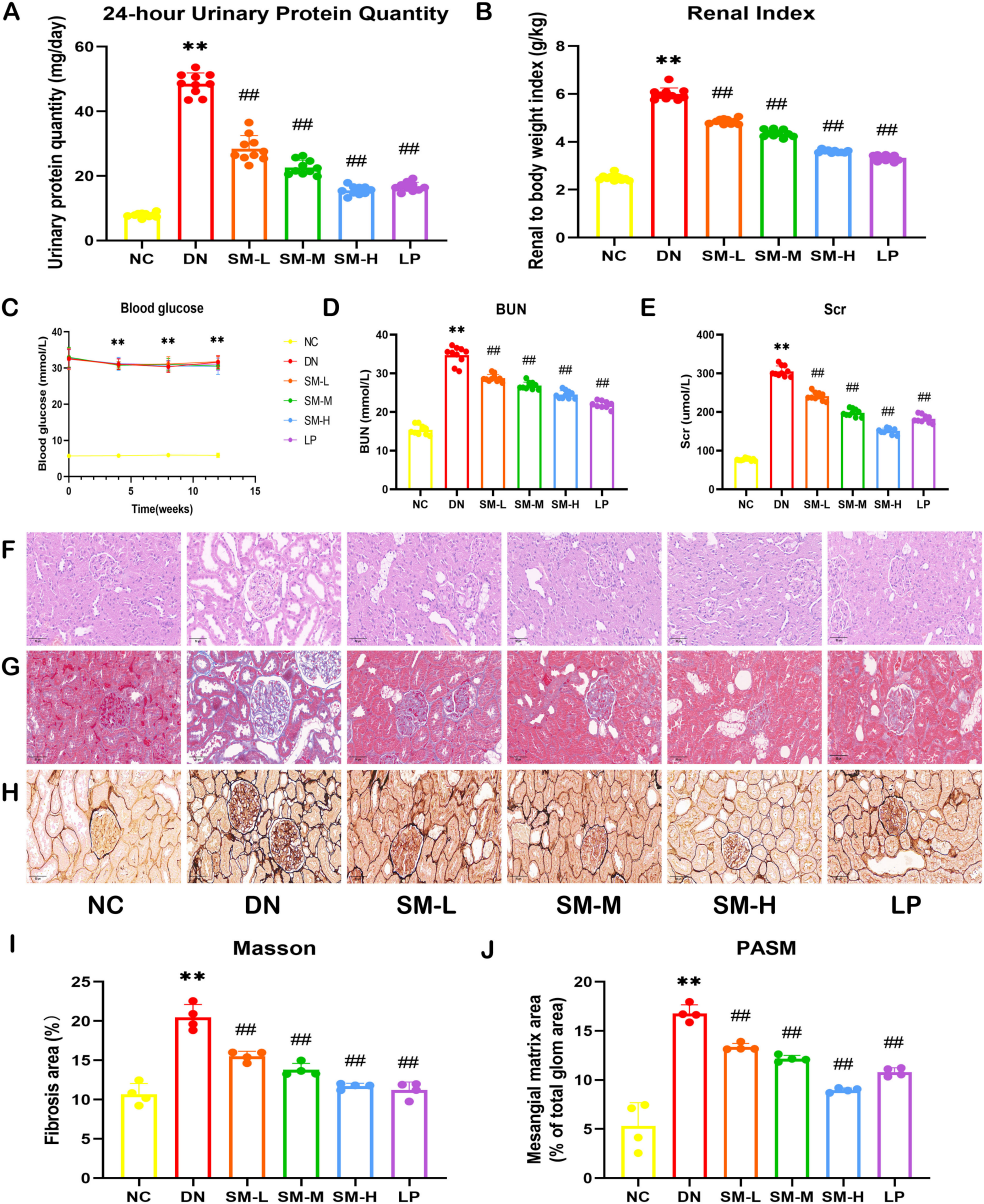
Effects of SM on DN rat. **(A)** 24-h-urine protein levels. **(B)** Renal Index. **(C)** Blood glucose levels. **(D)** BUN levels. **(E)** Scr levels. **(F)** Representative images of H&E staining (200 ×; scale bar = 50 μm). **(G)** Representative images of PASM staining (400 ×; scale bar = 20 μm). **(H)** Representative images of Masson staining (400 ×; scale bar = 20 μm). **(I)** Mesangial matrix area. **(J)** Fibrosis area. The data are X ± SEM. ** *p* < 0.01 *vs*. normal control (NC) group; ^##^ *p* < 0.01 *vs*. diabetic nephropathy (DN) group.

The findings indicated a significant increase in 24-hour urinary protein and kidney weight/body weight ratio in DN rats compared to NC rats, while DN rats treated with SM/LP exhibited a reduction in these parameters (**Figures 4A, B**). Additionally, the M group, after STZ interventions, displayed blood glucose levels exceeding 16.7 mmoL/L (**Figure 4C**). Furthermore, following a 12-week period of SM treatment, our study revealed that there was no statistically significant variance in blood glucose levels between the DN group and the SM group, suggesting that SM did not confer a notable advantage in terms of blood glucose regulation in DN rats. Analysis of **Figures 4D, E** indicated that levels of BUN and Scr were markedly elevated in the DN group. Still, following treatment with the drug, there was a significant decrease in Scr and BUN levels in the SM/LP-treated groups. Subsequently, the impact of SM on morphological alterations in DN rats was assessed through HE, MASSON and PAS staining (**Figures 4F–J**). Our findings indicate that SM mitigated renal injury in DN rats dose-dependently. Furthermore, the therapeutic efficacy of SM was comparable to that of LP, a commonly prescribed medication for the clinical management of DN. HE, PAS and MASSON staining confirmed these findings, demonstrating a reduction in glycogen deposition in the glomeruli of DN rats following SM/LP treatment. Notably, the improvements were more pronounced in the SM-H and LP groups. These results provide compelling evidence that SM possesses significant potential for treating DN.

#### 3.3.2 Effects of SM on mitochondrial morphology and function in DN rats

**Figure 5:**
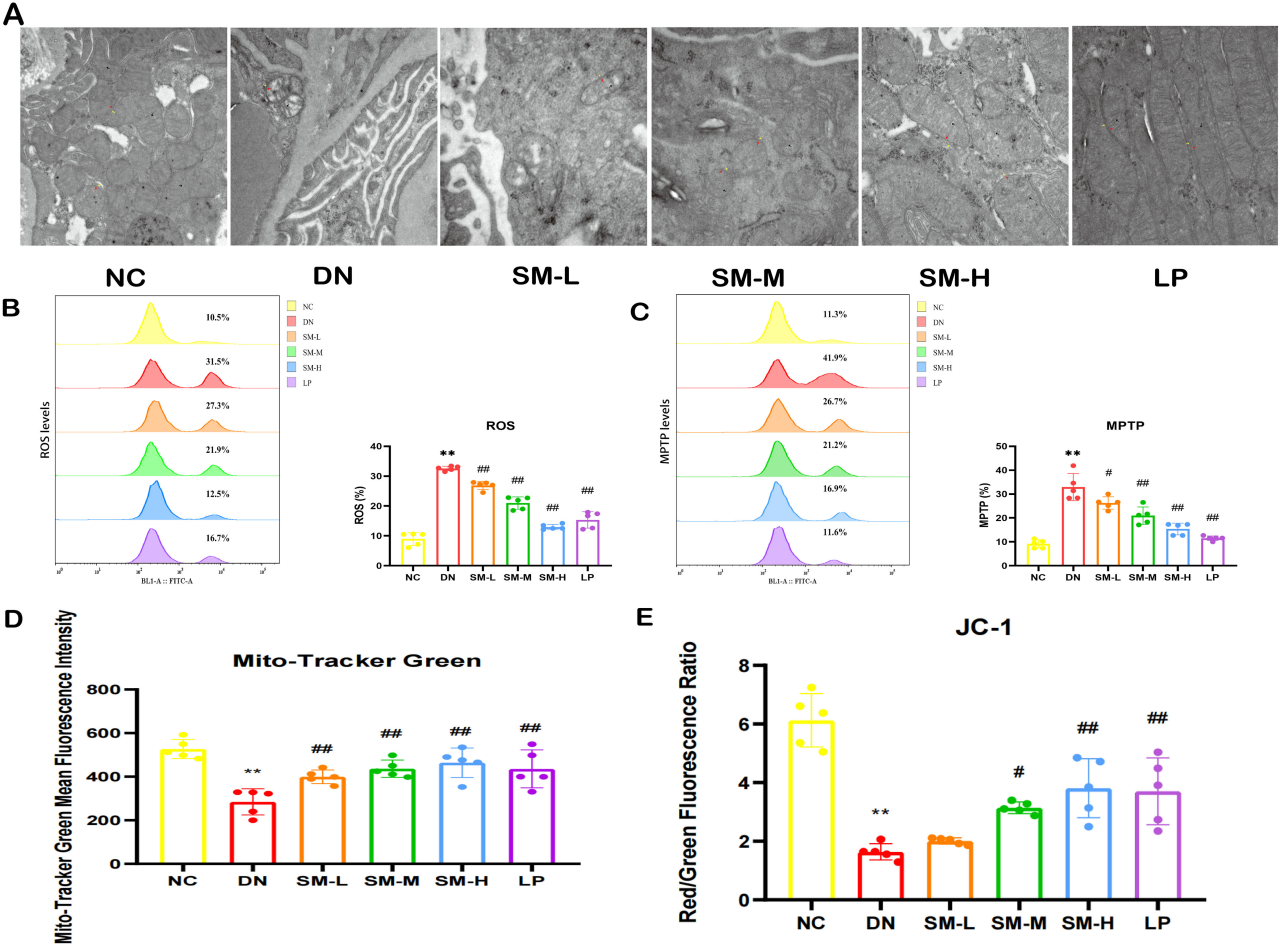
Effects of SM on mitochondrial morphology and function in DN rats. **(A)** Representative images of the ultrastructure of mitochondria on renal tissue (× 15000, Scale bar = 500 nm). Yellow arrows represent the outer mitochondrial membrane, red arrows represent the inner mitochondrial membrane, and black arrows represent cristae. **(B)** Flow diagrams of ROS (n = 5). **(C)** Flow diagrams of MPTP (n = 5). **(D)** Quantitative analysis of mitochondrial content by Mito Tracker Green (n = 5). **(E)** Quantitative analysis of MMP (n = 5). The data are X ± SEM. ** *p* < 0.01 *vs*. normal control (NC) group; ^#^ *p* < 0.05 *vs*. diabetic nephropathy (DN) group; ^##^ *p* < 0.01 *vs*. diabetic nephropathy (DN) group.

The alterations in mitochondrial ultrastructure were elucidated through TEM. The TEM images revealed that the mitochondrial morphology and structure in the kidney tissues of DN rats and NC rats were predominantly normal. However, compared to the NC group, the DN group exhibited decreased mitochondrial volume, the darker coloration of mitochondria, ruptured mitochondrial membranes, and reduced cristae. SM/LP-treated groups were improved when compared with those in the DN group, and the most noticeable improvement was found in the SM-H and LP groups (**Figure 5A**).

Mitochondrial dysfunction was identified by decreased mitochondrial content, elevated ROS production, heightened MPTP opening, and diminished MMP.

Mitotracker Green indicated changes in mitochondrial content, DCFH-DA staining assessed changes in ROS, JC-1 probes determined changes in MMP, and Calcein AM staining suggested the degree of MPTP opening. In the DN group, it was observed that excessive ROS production correlated with reduced MMP, increased MPTP opening, and significantly decreased mitochondrial content (**Figures 5B–E**). However, treatment with SM-H/LP partially reversed the decline in MMP and mitochondrial content, inhibited MPTP opening, and reduced ROS production (**Figures 5B–E**). In summary, SM-H treatment mitigated mitochondrial damage.

#### 3.3.3 SM ameliorated STZ-induced inflammatory response in the rats with DN

**Figure 6:**
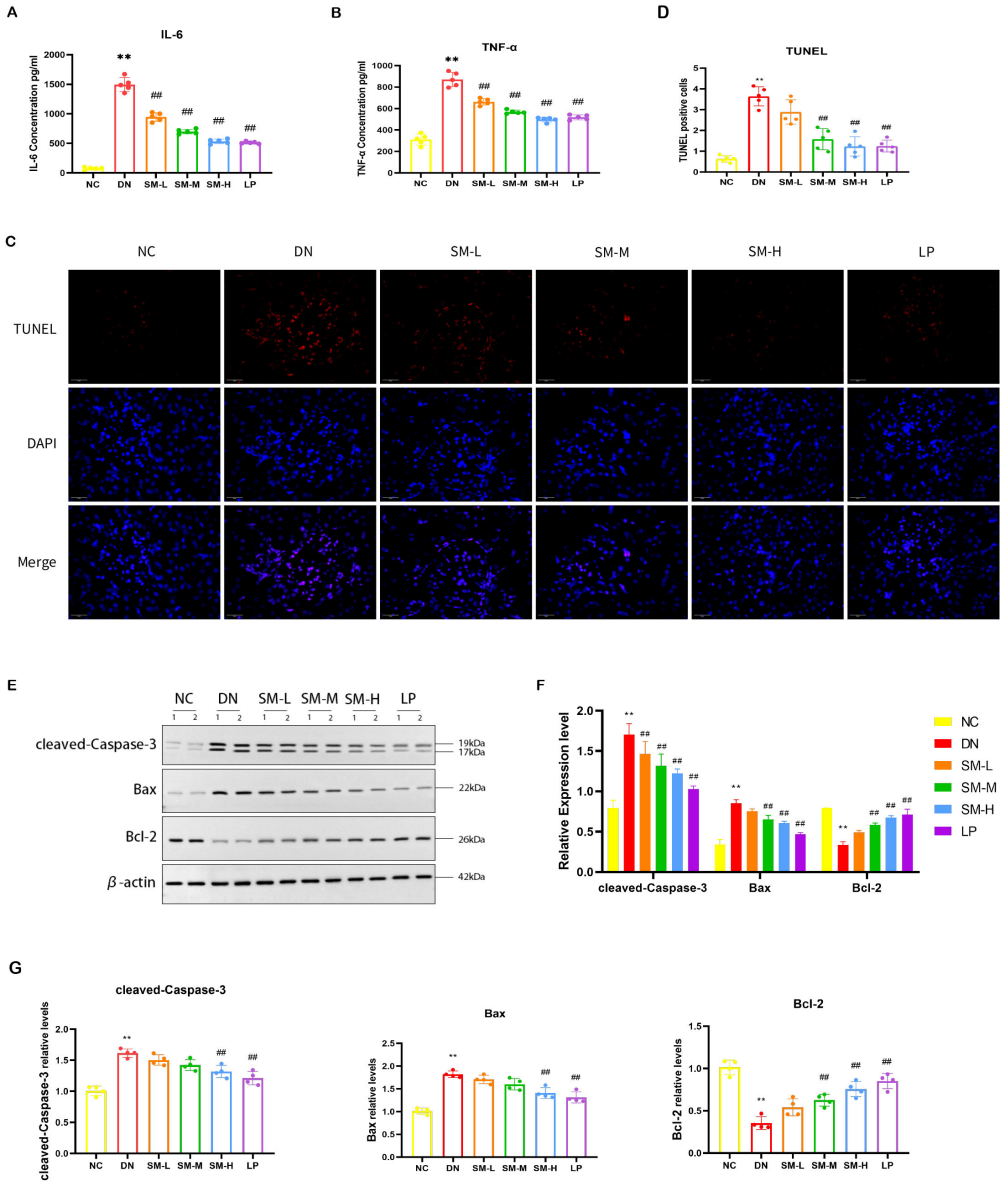
SM administration improves renal inflammation response and apoptosis in DN rats. **(A, B)** IL-6 and TNF-α levels in kidney tissue were detected by ELISA (n = 5). **(C)** Representative micrographs of TUNEL staining were given (200 ×; Scale bar = 50 μm). **(D)** Results of quantitative determination of TUNEL staining (n = 5). **(E)** The expression of cleaved-Caspase-3, Bax, and Bcl-2. β-actin was used as a loading control. **(F)** Results of quantitative determination of western blotting of cleaved-Caspase-3, Bax, and Bcl-2 (n = 4). **(G)** PCR analysis of Caspase-3, Bax, Bcl-2, and β-actin (n = 4). The data are X ± SEM. ** *p* < 0.01 *vs*. normal control (NC) group; ^##^ *p* < 0.01 *vs*. diabetic nephropathy (DN) group.

To assess the impact of SM on the inflammatory response in DN *in vivo*, the expression levels of inflammatory factors in renal tissues post-modeling were analyzed using ELISA. The findings indicated a notable upregulation and enhanced expression of IL-6 and TNF-α in the DN group (*p* < 0.05). Subsequent intervention with SM and LP significantly mitigated this effect (*p* < 0.05). Consequently, SM demonstrates efficacy in suppressing the inflammatory response in DN, as evidenced by the decreased expression of IL-6 and TNF-α, suggesting its potential for inhibiting DN via the inflammatory signaling pathway (**Figures 6A, B**).

#### 3.3.4 SM ameliorated STZ-induced apoptosis in the rats with DN

TUNEL staining showed that apoptotic cells increased in the DN group, while apoptotic cells decreased after SM-M and SM-H treatment (**Figures 6C, D**). Moreover, western blotting showed that cleaved-caspase-3 and Bax were up-regulated in the DN group compared with the NC group, in contrast to the down-regulation of Bcl-2. The administration of SM-H/LP treatment reduced the downregulation of anti-apoptotic proteins and the inhibition of the upregulation of pro-apoptotic factors. Consistent with the findings of protein blotting, PCR analysis demonstrated higher levels of cleaved-caspase3 and Bax expression in the DN group compared to the NC group (*p* < 0.01). In contrast, Bcl-2 expression was lower in the DN group (*p* < 0.01). Treatment with SM-H effectively modulated this specific expression pattern. These results indicate that SM-H may mitigate DN by suppressing apoptosis (**Figures 6C–G**).

#### 3.3.5 Regulation of PI3K/AKT/mTOR signaling pathway and JAK2/STAT3 signaling pathway by SM

**Figure 7:**
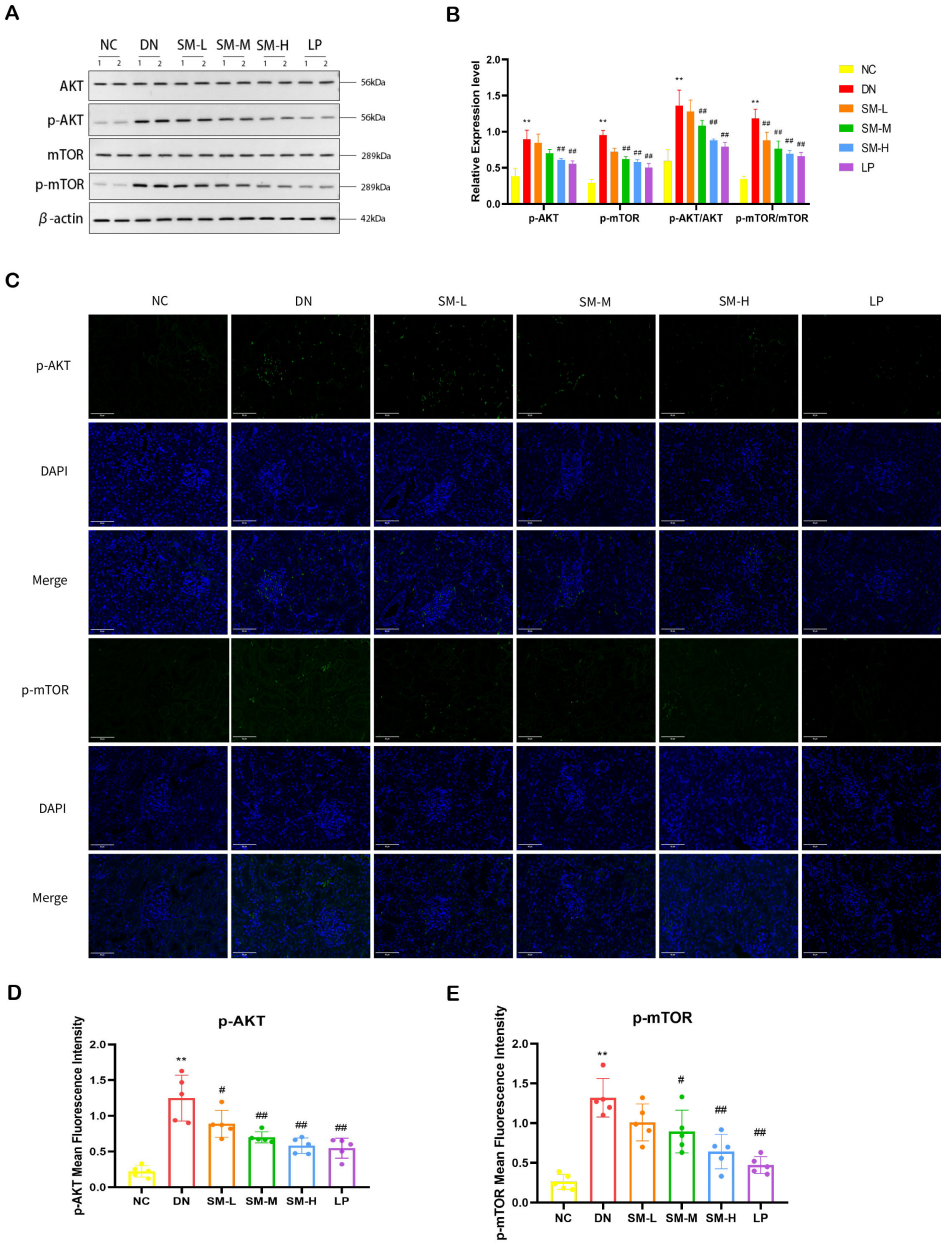
Effect of SM on PI3K/AKT/mTOR signaling pathway expression in rats with DN kidney tissues. **(A)** The expression of AKT, p-AKT, mTOR, and p-mTOR. β-actin was used as a loading control. **(B)** Quantitative determination of p-AKT, p-AKT, and p-mTOR’s relative abundances (n = 4). **(C)** Immunofluorescence analysis that shows the abundance and distribution of p-AKT and mTOR. Representative micrographs were given (200 ×; Scale bar = 50 μm). **(D, E)** The graph below shows quantitation data of an average of 5 independent p-AKT and p-mTOR immunofluorescence staining. The data are X ± SEM. **p < 0.01 *vs*. normal control (NC) group; ^#^ *p* < 0.01 vs. diabetic nephropathy (DN) group; ^##^p < 0.01 *vs*. diabetic nephropathy (DN) group.

Western blot analysis was used to semi-quantitatively assess the expression levels of four proteins: AKT, p-AKT, mTOR, and p-mTOR. The findings indicated a notable increase in the phosphorylated protein expression of AKT and mTOR in DN rats. At the same time, no significant differences were observed in the total levels of AKT and mTOR compared to the NC group (**Figures 7A, B**). The ratios of p-AKT/total AKT protein and p-mTOR/total mTOR protein were calculated, revealing a significant increase in the ratios of p-AKT/AKT and p-mTOR/mTOR in the DN group. As anticipated, phosphorylated AKT and mTOR protein levels were notably reduced in the treatment groups following 12 weeks of SM-H/LP administration (*p* < 0.01). IF analysis revealed significant expression of p-AKT and p-mTOR in the glomeruli and tubulointerstitium of the DN group, while a decreasing trend in p-AKT and p-mTOR expression was observed in the rats in the SM-M, and SM-H groups (**Figures 7C–E**).

**Figure 8:**
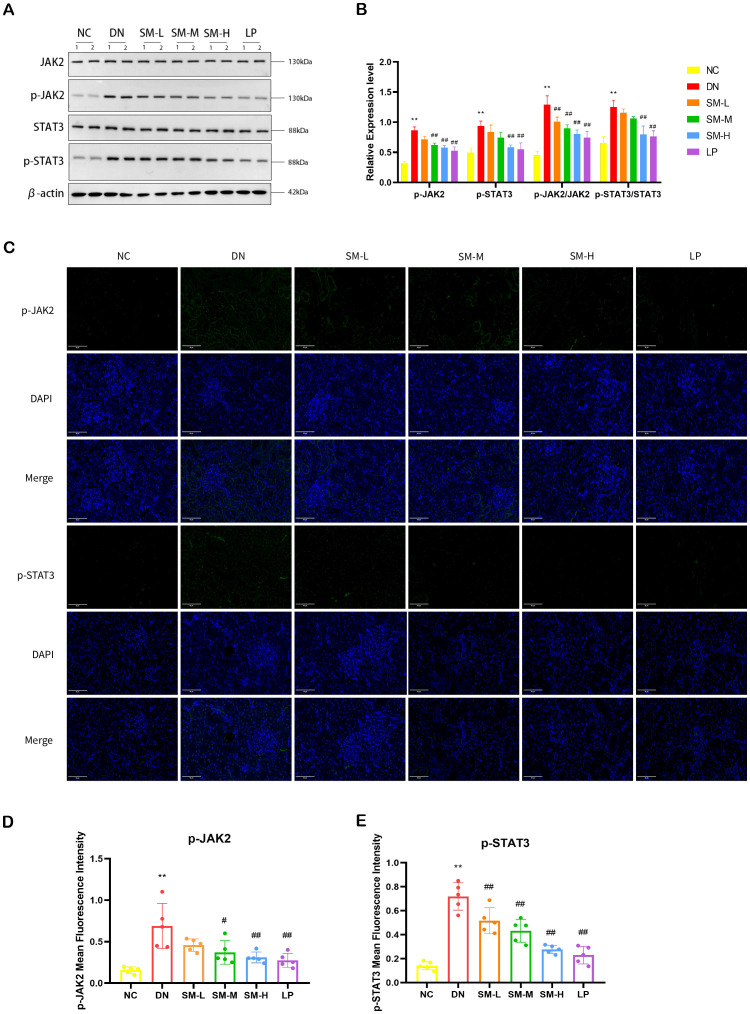
Effect of SM on JAK2/STAT3 signaling pathway expression in rats with DN kidney tissues. **(A)** The expression of JAK2, p-JAK2, STAT3, and p-STAT3. β-actin was used as a loading control. **(B)** Quantitative determination of the relative abundances of JAK2, p-JAK2, STAT3, and p-STAT3 (n = 4). **(C)** Immunofluorescence analysis that shows the abundance and distribution of p-JAK2 and p-STAT3. Representative micrographs were given (200 ×; Scale bar = 50 μm). **(D, E)** The graph below shows quantitation data of an average of 5 independent p-AKT and p-mTOR immunofluorescence staining. ** *p* < 0.01 *vs*. normal control (NC) group; ^#^ *p*< 0.01 vs. diabetic nephropathy (DN) group; ^##^ *p* < 0.01 *vs*. diabetic nephropathy (DN) group.

In order to further explore the potential relationship between the anti-inflammatory and anti-apoptotic effects of SM on DN and the JAK2/STAT3 signaling pathway, Western blot analysis was conducted to assess the levels of JAK2, STAT3, and phosphorylation in rat kidney tissues. The experiment results indicated that, compared to the NC group, there was no significant difference in the total levels of JAK2 and STAT3 proteins in rat kidney tissues. Of the DN group. However, there was a substantial increase in the relative expression of p-JAK2/JAK2 and p-STAT3/STAT3, suggesting a potential activation of the JAK2/STAT3 pathway in DN. Compared to the DN group, the treatment group exhibited minimal changes in the total protein expression levels of JAK2 and STAT3 in renal tissues (*p* < 0.01). However, there was a noticeable reduction in the protein expression of p-JAK2 and p-STAT3 in the SM-H and LP groups, which demonstrated significant therapeutic effects as depicted in **Figures 8A, B**. Immunofluorescence analysis revealed a substantial expression of p-JAK2 and p-STAT3 in glomeruli and tubulointerstitium of the DN group, whereas the SM-H showed a marked decrease in the expression of p-JAK2 and p-STAT3 (**Figures 8C–E**).

In conclusion, SM-H can regulate the PI3K/AKT/mTOR signaling pathway and the JAK2/STAT3 signaling pathway to inhibit the inflammatory response and apoptosis.

### 3.4 Verification of the interaction between identified bioactive compounds and HUB targets via molecular docking

**Figure 9:**
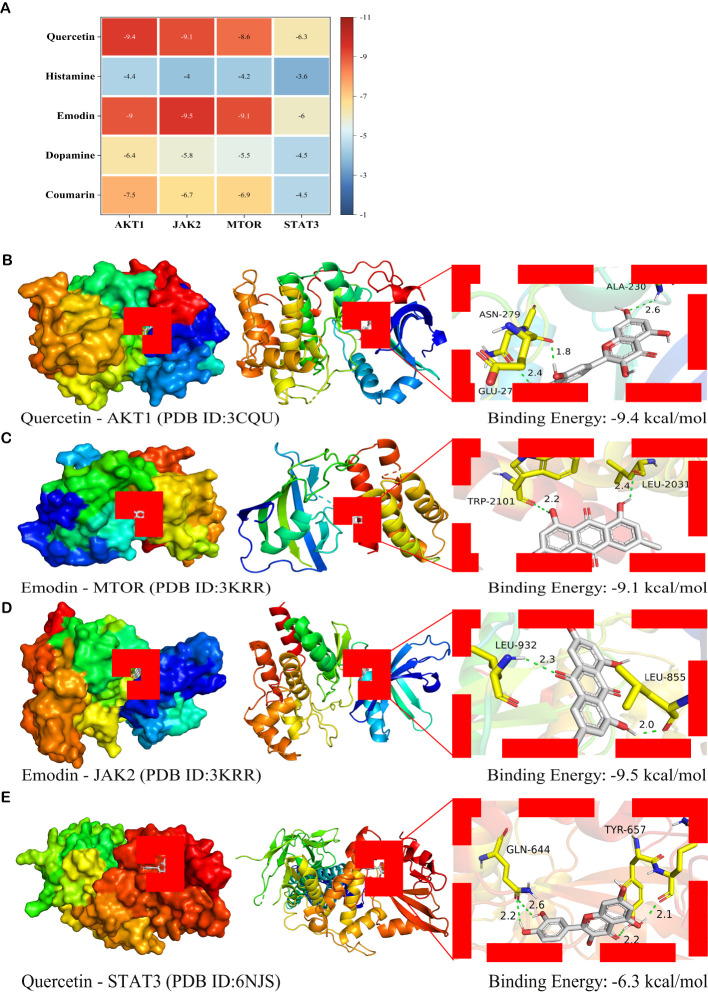
Molecular docking analysis of the main active ingredients with essential proteins **(A)** Heat map of binding energy (kcal/mol). The vertical axis represents the bioactive ingredient of SM, and the horizontal axis represents the core target protein receptor. Each value represents the binding energy of molecular docking; the darker the color, the greater the absolute value of the binding energy. **(B)** Docking analysis of Quercetin with AKT1 receptor. **(C)** Docking analysis of Emodin with mTOR receptor. **(D)** Docking analysis of Emodin with JAK2 receptor. **(E)** Docking analysis of Quercetin with STAT3 receptor. Green dashed lines, interacting hydrogen bonds.

The mean affinity value of 20 groups was -6.55 kcal/mol and 14 groups had affinity ≤ -7.00 kcal/mol. Thus, the therapeutic effect of SM on DN was verified at the molecular docking level (**Figure 9A**). Additionally, the average binding energy with AKT1 was found to be the lowest among the five principal bioactive constituents (**Figure 9A**). The docking patterns of the four combinations of ‘target protein-active molecule’ with small binding energies were graphed, as depicted in **Figures 9B–E**. This finding provides additional evidence that the primary active constituents of SM interact with essential targets such as AKT1, mTOR, and JAK2, among others, and exhibit strong affinity towards these critical targets. This observation suggests that the primary active constituents of SM may have therapeutic potential for the treatment or amelioration of DN through interactions with core targets.

## 4 Discussion

Initially, a total of 1281 constituents of SM ethanol extract were identified using the UPLC-ESI-MS/MS coupling technique. Subsequently, three databases were consulted to collect 3760 DN-related target genes: GeneCards, OMIM, and DisGeNET. Also, 1226 DEGs were identified from the GEO database through RNA sequencing analyses in this investigation. GSEA prioritizes gene sets with shared biological functions, chromosomal locations, and expression regulation within the gene matrix. To investigate the precise pathogenic mechanism of SM in DN, the GSE30528 dataset was chosen for additional examination. The GSEA findings of this study revealed enrichment of 50 SM-related gene sets in DN, notably including IL6/JAK-STAT3 signaling, inflammation response, oxidative phosphorylation, epithelial-mesenchymal transition, UA response DN, TGF-β signaling, etc. In addition, 2025 SM putative target genes were cross-combined with 1226 DEGs and 3760 DN-associated target genes, resulting in a total of 126 overlapping genes. These 126 target genes were considered potential therapeutic targets for the improvement of DN by SM. Then, we constructed a PPI network of overlapping genes and screened 15 key targets, including CCL2, PTPRC, EGF, IGF1, SPP1, CD36, VWF, COL1A1, CCR2, CD40LG, CSF1R, JAK2, CCR5, CXCR2, and MMP1. One-third of the nodes are primarily associated with the PI3K-AKT signaling pathway in diabetic complications. The majority of the HUB genes target cellular signal transduction factors. Through the integration of GSEA and KEGG analyses, JAK2 is identified as a pivotal gene for the treatment of DN. The significant diagnostic utility of JAK2 for DN was confirmed through ROC curve analysis on the validation set (GSE96804). JAK2 is a non-receptor tyrosine kinase that is a crucial component in cytoplasmic signaling cascades initiated by cytokine receptors. Upon activation by cytokines, JAK2 initiates receptor phosphorylation and subsequently recruits and phosphorylates signal transducer and activator of transcription (STAT) proteins to facilitate intracellular signaling (28, 29). Research has indicated that various immune cytokines activate JAK, essential in cellular immunity, cell proliferation, differentiation, and apoptosis (30, 31). Investigations have revealed elevated levels of JAK2 expression in the glomerular and tubulointerstitial regions of patients with DN compared to controls, indicating a potential association with inflammation, immune injury, apoptosis, and other mechanisms of DN (32, 33). Subsequent studies have demonstrated that inhibiting the activity of JAK2 can lead to improved renal function and disease alleviation in DN patients (33, 34). These studies suggest that JAK2 is involved in specific pathogenic mechanisms of DN and could be a potential therapeutic target for DN.

The network analysis findings presented a broader and more holistic understanding of the therapeutic mechanism of tonifying the kidney and activating blood in the treatment of DN. An enrichment analysis was conducted on overlapping genes to further elucidate the potential molecular mechanisms of SM in treating DN. The KEGG results indicated that the PI3K-AKT signaling pathway, with a lower P value and strong connections to other pathways, was significantly enriched with the highest number of genes in the HUB genes, suggesting it is the most crucial pathway for SM in treating DN. The findings of these enrichment analyses indicate that the potential mechanisms underlying the therapeutic effects of SM on DN may involve the modulation of inflammatory responses, regulation of mitochondrial dysfunction, and inhibition of apoptosis.

To corroborate these bioinformatics results, a series of experimental studies were undertaken. Initially, the impact of SM on renal function and histopathology in rats with DN induced by a combination of HSHFD and STZ was examined. Key diagnostic biomarkers of DN, including blood glucose levels, serum creatinine, blood urea nitrogen, and 24-hour urinary protein excretion, were assessed to evaluate renal function (35).

Furthermore, pre-network pharmacology and bioinformatics analyses suggested that SM-H’s anti-DN mechanism of action may involve the regulation of the PI3K/AKT/mTOR axis and JAK2/STAT3 axis. Subsequent *in vivo* experiments demonstrated that the therapeutic effect of SM-H (6 g/kg/d) on DN was stable and significant. In addition, renal tissue analysis through PCR, Western blotting, and immunofluorescence techniques indicated that the compound SM-H suppressed the expression of IL-6 and TNF-α, as well as Bax and BCL-1 while also reducing the activities of p-AKT, p-mTOR, p-JAK2, and p-STAT3, and increasing levels of cleaved-caspase 3.

Mitochondria are widely recognized as pivotal regulators of energy metabolism and apoptosis in organisms. The functionality of mitochondria is governed by Bcl-2 family proteins, which consist of anti-apoptotic proteins such as Bcl-2 and Bcl-xL, as well as pro-apoptotic proteins like Bad, Bid, and Bax (36, 37). Furthermore, upon cellular stimulation, the excessive generation of ROS hinders the release of cytochrome C and other pro-apoptotic factors into the cytoplasm by impeding the extensive opening of the mitochondrial MPTP. In contrast, pro-apoptotic proteins reduce MMP, leading to heightened outer mitochondrial membrane permeability and subsequent liberation of apoptotic factors, such as cytochrome C, into the cytoplasm. Upon release, cytochrome C triggers the activation of apoptotic protease activating factor-1 (Apaf-1), forming apoptotic bodies composed of cytochrome C, Apaf-1, and pro-caspase-9. These bodies subsequently cleave pro-caspase-9 into cleaved-caspase-9, which then cleaves pro-caspase-3 into cleaved-caspase-3. The cleaved-caspase-3 enzyme hydrolytically cleaves various regulatory and structural proteins, thereby initiating the process of apoptosis (38–40). Various research studies have demonstrated the potential of Bcl-2 as a biomarker for renal function and its role in apoptosis during the progression of DN (41). In a survey by Xue-Qi Liu et al., it was demonstrated that wogonin treatment improved renal histopathological changes in high glucose-induced podocyte injury and STZ-induced diabetic mice by targeting the modulation of Bcl-2-mediated anti-autophagy and anti-apoptotic effects (42).

PI3K is commonly activated through ligand binding, leading to the activation of AKT, which subsequently phosphorylates mTOR. This activation of mTOR plays a crucial role in various cellular processes such as mitochondrial energy metabolism, inflammation, oxidative stress, apoptosis, epithelial-mesenchymal transition, and autophagy-related signaling pathways in DN, contributing significantly to the progression of the disease (43–45). Saikosaponin-A has been proposed as a potential treatment for mitigating mitochondrial dysfunction and apoptosis by inhibiting the PI3K/AKT signaling pathway (46). Furthermore, Yih-Gang Goan et al. demonstrated that targeted suppression of the PI3K/AKT/mTOR pathway can impede apoptosis and mitochondrial dysfunction (47). Emerging evidence suggests that an imbalance between pro- and anti-inflammatory factors in the kidney plays a significant role in the development of DN (48, 49). Pro-inflammatory factors, such as TNF-α and IL-6, normally exist in equilibrium. However, prolonged hyperglycemia and hyperlipidemia can lead to increased expression of ROS and pro-inflammatory factors in the body, damaging renal tubular epithelial cells and disrupting kidney glomerular function (50).

This can lead to the proliferation of thylakoid cells, renal fibrosis, or glomerulosclerosis. The activation of the JAK/STAT pathway has been implicated in the signaling of cytokines such as IL-6, TNF-α, Bax, and BCL-1, which in turn activate the expression of genes related to inflammatory infiltration, apoptosis of immune response, and cell proliferation, thus promoting the development of DN (51, 52). JAK2 and STAT3 are the most frequently studied isoforms associated with DN. Maolin Zhu et al. discovered that targeting the JAK2/STAT3 signaling pathway resulted in decreased levels of IL-6, ICAM-1, Bcl2, and Bax in renal tissues of rats with STZ-induced DN, leading to a reduction in inflammatory response, apoptosis, mitigation of pathological damage to the kidneys, and enhancement of renal function (53). Yu-Li Shen et al. demonstrated that herbal extracts could mitigate the inflammatory response and protect renal function in DN rats by targeting and inhibiting the CXCL6/JAK/STAT3 pathway (54).

Furthermore, research has shown that Chinese medicine can reduce proteinuria in mice with chronic renal failure by inhibiting the JAK2/STAT3 and PI3K/AKT signaling pathways (35). These findings collectively suggest that the protective effects of SM are attributed to the suppression of the PI3K/AKT/mTOR axis and the JAK2/STAT3 axis, which inhibit apoptosis and inflammatory responses, regulate mitochondrial dysfunction, and improve renal pathological damage.

Finally, the study confirmed the interactions of the identified compounds with the PI3K/AKT/mTOR and JAK2/STAT3 signaling pathways through molecular docking analysis. The results of the docking analysis suggested that multiple compounds exhibit stable binding to the validated targets. Specifically, the primary active components of SM, including Coumarin, Emodin, and Quercetin, demonstrated high affinity for these key targets, indicating their potential significance in the treatment of DN with SM. A literature search was conducted to investigate the potential efficacy of key compounds in protecting renal function and treating DN. Subsequent *in vivo* and *in vitro* studies demonstrated that quercetin could mitigate renal injury in DN by modulating the Nrf2/HO-1 signaling pathway, leading to improvements in blood creatinine levels and inhibition of ferroptosis in renal tubular epithelial cells (55). Additionally, the coumarin derivative has been shown to function as an Nrf2 activator, effectively inhibiting high glucose-induced oxidative stress and fibrosis in renal mesangial cells and exerting a protective effect on glomeruli (56). Current evidence indicates that treating a KK-Ay mouse model with DN using Emodin for 8 weeks significantly reduces urinary albumin, Scr, and BUN levels (57). Additionally, this treatment improves thylakoid matrix expansion, glycogen storage, and pedunculated synaptic fusion by inhibiting the activation of the PERK/eIF2α signaling pathway from mitigating apoptosis (57). These findings have been corroborated by *in vitro* studies. Further validation experiments are necessary to elucidate the potential roles and mechanisms of the monomers above. In general, the bioinformatics results were accurate and practical.

## 5 Conclusion

**Figure 10:**
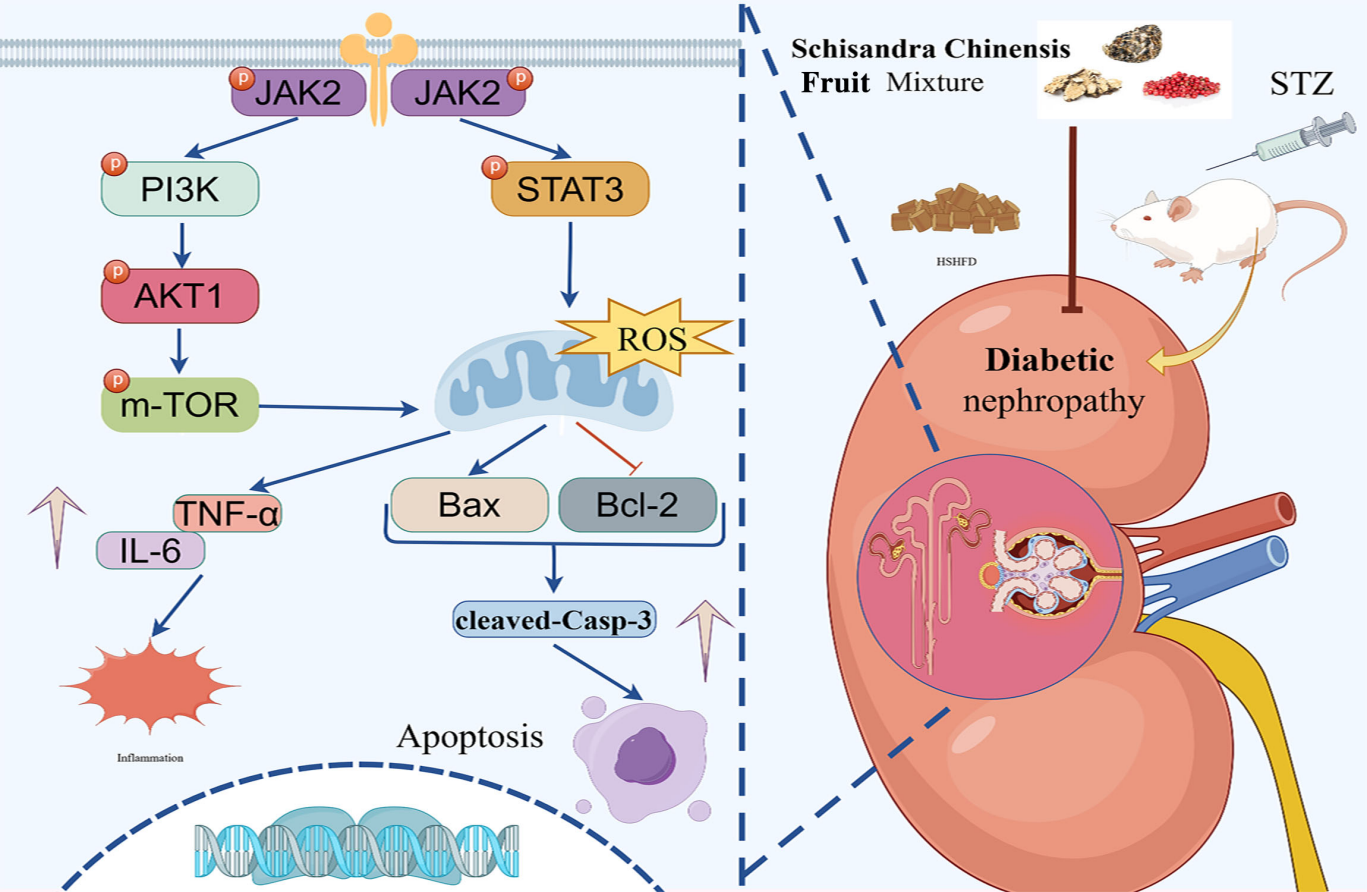
The illustration of the JAK2/STAT3 and PI3K/AKT signaling pathways implicated in the protective effect of SM on kidneys injured by DN. SM stimulated the JAK2/STAT3 pathway, subsequently stimulating the PI3K/AKT/mTOR signaling cascade, which resulted in elevated Bcl-2 levels and reduced Bax, IL-6, TNF-α, and cleaved caspase-3, so inhibiting apoptosis and inflammation.

To sum up, our research verified that the JAK2/STAT3 and PI3K/AKT/mTOR pathways contribute to the anti-DN effects of SM (**Figure 10**).

## Data Availability

The original contributions presented in the study are included in the article/**Supplementary Material**. The GEO data provided in this study are deposited in the GEO repository under accession number GSE30528.The data presented in the study are publicly available. This data can be found here: https://github.com/1977426071/20250212. Further inquiries can be directed to the corresponding authors.

## Glossary

AKT: protein kinase B
AUC: area under the curve
ACEi: angiotensin-converting enzyme inhibitors
ARBs: angiotensin receptor blockers
Bcl-2: B-cell lymphoma-2
Bax: bcl-2-associated X protein
BUN: blood urea nitrogen
DEGs: differentially expressed genes
DN: diabetic nephropathy
ELISA: enzyme-linked immunosorbent assay
GO: gene ontology
H&E: hematoxylin-eosin
IL-6: interleukin 6
IF: immunofluorescence
JAK2: Janus kinase 2
KEGG: kyoto encyclopedia of genes and genomes
mTOR: mammalian target of rapamycin
MPTP: mitochondrial membrane permeability transition pore
MMP: mitochondrial membrane potential
NS-MRAs: non-steroidal mineralocorticoid receptor antagonists
PASM: periodic acid-silver
PI3K: phosphatidylinositol 3 kinase metheramine
PPI: protein-protein interactions
ROS: reactive oxygen species
SM: Schisandra chinensis Fruit Mixture
STAT3: signal transducer and activator of transcription 3
Scr: serum creatinine
STZ: streptozotocin
SGLT2: sodium-glucose cotransporter-2
TCM: traditional Chinese medicine
TCMSP: TCM systems pharmacology
TNF-α: tumor necrosis factor alpha
UPLC-ESI-MS/MS: ultrahigh-performance liquid chromatography-electrospray ionization-tandem mass spectrometry
PPI: protein-protein interaction.
