# Supplementary material for: Integrated UPLC-ESI-MS/MS, network pharmacology, and transcriptomics to reveal the material basis and mechanism of Schisandra chinensis Fruit Mixture against diabetic nephropathy
Source: Front Immunol. 2025 Feb 19;15:1526465. doi: 10.3389/fimmu.2024.1526465 (PMC11879837; doi:10.3389/fimmu.2024.1526465)
Supplement: Supplementary file 1 [file Table1.docx]

**Integrated UPLC-ESI-MS/MS, network pharmacology, and transcriptomics to reveal the material basis and mechanism of Schisandra chinensis Fruit Mixture against diabetic nephropathy**

Yuan-Yuan Deng^1,2,3,4+^, Xin-Yu Ma^1+^, Peng-Fei He^1,4+^, Zheng Luo^5^, Ni Tian^2^, Shao-Ning Dong^2^, Sai Zhang^1^, Jian Pan^2^, Peng-Wei Miao^2^, Xiang-Jun Liu^2^, Cui Chen^2^, Peng-Yu Zhu^2^，Bo Pang^2,6^, Jing Wang^2,6^, Li-Yang Zheng^2,6^, Xin-Kun Zhang^2^, Min-Ying Zhang^7^ and Mian-Zhi Zhang^1,2#^

**∗ Corresponding author**

E-mail addresses: zhangmianzhi@vip.sina.com (M. Zhang).

1 These authors contributed equally to this work.**Supplementary Table 1**

**Component herbs of SM**

| **Chinese name** | **Pharmaceutical**  **name** | **Family and plant parts**  **used** | **Botanical plant name** | **Amount inpreparation (g / %)** |
| --- | --- | --- | --- | --- |
| Wuweizi | Schisandrae chinensis  fructus | Magnoliaceae; fruit | Schisandra chinensis (Turcz.) Baill. | 30g / 50% |
| Chuanxiong | Ligusticum chuanxiong Hort. | Apiaceae; root and  rhizome | Ligusticum wallichii Franch. | 20g / 33.3% |
| Muli | Ostreae concha | Ostreidae; shell | Crassostrea gigas Thunberg Crassostrea talienwhanensis | 10g / 16.7% |

**Supplementary Table 2**

**Detailed information on key targets**

| **Protein** | **PDB ID** | **Center (X, Y, Z)** | **Size (X × Y × Z)** |
| --- | --- | --- | --- |
| AKT1 | 3CQU | 6.0, 0.0, 19.0 | 26.0 × 26.0 ×24.0 |
| JAK2 | 3KRR | 18.6, 18.2,5.3 | 21.0 × 24.0 × 9.6 |
| mTOR | 5GPG | -18.9, 2.2, 9.6 | 20.0 × 23.0 × 24.0 |
| STAT3 | 6NJS | 27.0, 51.0, 8.0 | 31.0 × 32.0 × 37.0 |

**Supplementary Table 3**

**PCR Reaction Procedure**

| **Loop Steps** | **Temperature（℃）** | **Reaction Time** | **Number of cycles (times)** |
| --- | --- | --- | --- |
| 1 | 95℃ | 5 min | 1 |
| 2 | 95℃ | 10 sec | 2-4 step cycle 40 |
| 3 | 58℃ | 20 sec |  |
| 4 | 72℃ | 20 sec |  |
| 5 | 95℃ | 15 sec | 1 |
| 6 | 60℃ | 60 sec | 1 |
| 7 | 95℃ | 15 sec | 1 |

**Supplementary Table 4**

**Primer sequence list**

| **Gene name** | **Primer** | **Primer sequence** | **Product length / bp** |
| --- | --- | --- | --- |
| β-actin | F | CTGAACGTGAAATTGTCCGAGA | 145bp |
|  | R | TTGCCAATGGTGATGACCTG |  |
| Caspase-3 | F | AGCACCTGGTTACTATTCCTG | 101bp |
|  | R | TAAATTCTAGCTTGTGCGCGTA |  |
| Bcl-2 | F | ACTTCTCTCGTCGCTACCGTC | 117bp |
|  | R | CCCCATCCCTGAAGAGTTCCT |  |
| Bax | F | AGGGTTTCATCCAGGATCGAGCA | 95bp |
|  | R | CAGCTTCTTGGTGGACGCATC |  |

**Supplementary Table 5**

**Active components of SM**

| **No.** | **Compounds** | **Formula** | **Q1 (Da)** | **Ionization model** | **Class I** |
| --- | --- | --- | --- | --- | --- |
| 1 | α-Hydroxycinnamic Acid* | C9H8O3 | 1.63E+02 | [M-H]- | Phenolic acids |
| 2 | p-Coumaroyltyramine | C17H17NO3 | 2.84E+02 | [M+H]+ | Alkaloids |
| 3 | p-Coumaroylputrescine | C13H18N2O2 | 2.35E+02 | [M+H]+ | Alkaloids |
| 4 | p-Coumaroylmalic acid | C13H12O7 | 2.79E+02 | [M-H]- | Phenolic acids |
| 5 | p-Coumaric acid methyl ester | C10H10O3 | 1.79E+02 | [M+H]+ | Phenolic acids |
| 6 | p-Coumaric acid ethyl ester | C11H12O3 | 1.93E+02 | [M+H]+ | Phenolic acids |
| 7 | p-Coumaric acid | C9H8O3 | 1.65E+02 | [M+H]+ | Phenolic acids |
| 8 | p-Coumaraldehyde | C9H8O2 | 1.47E+02 | [M-H]- | Phenolic acids |
| 9 | Zarzissine | C5H5N5 | 1.36E+02 | [M+H]+ | Alkaloids |
| 10 | Vomifoliol (Blumenol A) | C13H20O3 | 2.23E+02 | [M-H]- | Terpenoids |
| 11 | Vanillin; 4-Hydroxy-3-Methoxybenzaldehyde | C8H8O3 | 1.51E+02 | [M-H]- | Others |
| 12 | Vanillin acetate | C10H10O4 | 1.95E+02 | [M+H]+ | Phenolic acids |
| 13 | Vanillic acid methyl ester | C9H10O4 | 1.81E+02 | [M-H]- | Phenolic acids |
| 14 | Vanillic acid | C8H8O4 | 1.67E+02 | [M-H]- | Phenolic acids |
| 15 | Tyrosol; 4-Hydroxyphenylethanol | C8H10O2 | 1.37E+02 | [M-H]- | Phenolic acids |
| 16 | Trigonelline | C7H7NO2 | 1.38E+02 | [M+H]+ | Alkaloids |
| 17 | Tricin (5,7,4'-Trihydroxy-3',5'-dimethoxyflavone) | C17H14O7 | 3.31E+02 | [M+H]+ | Flavonoids |
| 18 | Terephthalic acid | C8H6O4 | 1.65E+02 | [M-H]- | Phenolic acids |
| 19 | Syringic acid | C9H10O5 | 1.97E+02 | [M-H]- | Phenolic acids |
| 20 | Syringetin | C17H14O8 | 3.45E+02 | [M-H]- | Flavonoids |
| 21 | Syringaresinol | C22H26O8 | 4.17E+02 | [M-H]- | Lignans and Coumarins |
| 22 | Syringaldehyde; 4-Hydroxy-3,5-Dimethoxybenzaldehyde | C9H10O4 | 1.83E+02 | [M+H]+ | Others |
| 23 | Swertiamarin | C16H22O10 | 3.75E+02 | [M+H]+ | Terpenoids |
| 24 | Sweroside | C16H22O9 | 3.59E+02 | [M+H]+ | Terpenoids |
| 25 | Stearamide | C18H37NO | 2.84E+02 | [M+H]+ | Alkaloids |
| 26 | Spermine | C10H26N4 | 2.03E+02 | [M+H]+ | Alkaloids |
| 27 | Spermidine | C7H19N3 | 1.46E+02 | [M+H]+ | Alkaloids |
| 28 | Skimmin (7-Hydroxycoumarin-7-O-glucoside) | C15H16O8 | 3.25E+02 | [M+H]+ | Lignans and Coumarins |
| 29 | Sinapoyl malate | C15H16O9 | 3.41E+02 | [M+H]+ | Phenolic acids |
| 30 | Sinapine | C16H24NO5+ | 3.10E+02 | [M]+ | Alkaloids |
| 31 | Sinapinaldehyde | C11H12O4 | 2.07E+02 | [M-H]- | Phenolic acids |
| 32 | Sinapic acid | C11H12O5 | 2.23E+02 | [M-H]- | Phenolic acids |
| 33 | Senkyunolide C* | C12H12O3 | 2.05E+02 | [M+H]+ | Others |
| 34 | Senkyunolide B* | C12H12O3 | 2.05E+02 | [M+H]+ | Others |
| 35 | Senkyunolide A | C12H16O2 | 1.93E+02 | [M+H]+ | Others |
| 36 | Scopoletin (7-Hydroxy-6-methoxycoumarin)* | C10H8O4 | 1.93E+02 | [M+H]+ | Lignans and Coumarins |
| 37 | Salicylic acid* | C7H6O3 | 1.37E+02 | [M-H]- | Phenolic acids |
| 38 | Quinolinic Acid | C7H5NO4 | 1.66E+02 | [M-H]- | Alkaloids |
| 39 | Quinoline-4-carboxylic acid | C10H7NO2 | 1.72E+02 | [M-H]- | Alkaloids |
| 40 | Quinine | C20H24O2N2 | 3.25E+02 | [M+H]+ | Alkaloids |
| 41 | Quercetin-3-O-alloside; Isohyperoside* | C21H20O12 | 4.65E+02 | [M+H]+ | Flavonoids |
| 42 | Quercetin | C15H10O7 | 3.03E+02 | [M+H]+ | Flavonoids |
| 43 | Pyrocatechol | C6H6O2 | 1.09E+02 | [M-H]- | Phenolic acids |
| 44 | Putrescine | C4H12N2 | 8.91E+01 | [M+H]+ | Alkaloids |
| 45 | Protocatechuic Acid Methyl Ester | C8H8O4 | 1.67E+02 | [M-H]- | Phenolic acids |
| 46 | Propyl 4-hydroxybenzoate | C10H12O3 | 1.79E+02 | [M-H]- | Phenolic acids |
| 47 | Piperidine | C5H11N | 8.61E+01 | [M+H]+ | Alkaloids |
| 48 | Pinoresinol* | C20H22O6 | 3.57E+02 | [M-H]- | Lignans and Coumarins |
| 49 | Pinocembrin (Dihydrochrysin) | C15H12O4 | 2.57E+02 | [M+H]+ | Flavonoids |
| 50 | Pimaric acid* | C20H30O2 | 3.01E+02 | [M-H]- | Terpenoids |
| 51 | Picein (4-Acetylphenyl-glucoside) | C14H18O7 | 2.97E+02 | [M-H]- | Phenolic acids |
| 52 | Phthalic anhydride | C8H4O3 | 1.49E+02 | [M+H]+ | Phenolic acids |
| 53 | Phthalic acid | C8H6O4 | 1.65E+02 | [M-H]- | Phenolic acids |
| 54 | Phloroglucinol; 1,3,5-Benzenetriol | C6H6O3 | 1.27E+02 | [M+H]+ | Phenolic acids |
| 55 | Phloretin | C15H14O5 | 2.73E+02 | [M-H]- | Flavonoids |
| 56 | Phenyl-beta-D-glucoside | C12H16O6 | 2.55E+02 | [M-H]- | Phenolic acids |
| 57 | Phenyl acetate | C8H8O2 | 1.35E+02 | [M-H]- | Phenolic acids |
| 58 | Phenethyl caffeate | C17H16O4 | 2.83E+02 | [M-H]- | Phenolic acids |
| 59 | Perillyl alcohol | C10H16O | 1.53E+02 | [M+H]+ | Terpenoids |
| 60 | Oxindole | C8H7NO | 1.34E+02 | [M+H]+ | Alkaloids |
| 61 | Octadecadienamide | C18H33NO | 2.80E+02 | [M+H]+ | Alkaloids |
| 62 | O-Phosphocholine | C5H15NO4P+ | 1.84E+02 | [M]+ | Alkaloids |
| 63 | O-Acetyl-L-carnitine | C9H17NO4 | 2.04E+02 | [M+H]+ | Alkaloids |
| 64 | Nootkatone | C15H22O | 2.19E+02 | [M+H]+ | Terpenoids |
| 65 | Nicotinic Acid Methyl Ester(Methyl Nicotinate) | C7H7NO2 | 1.38E+02 | [M+H]+ | Alkaloids |
| 66 | Naringenin (5,7,4'-Trihydroxyflavanone)* | C15H12O5 | 2.73E+02 | [M+H]+ | Flavonoids |
| 67 | N-Oleoylethanolamine | C20H39NO2 | 3.26E+02 | [M+H]+ | Alkaloids |
| 68 | N-Hydroxypipecolic acid | C6H11NO3 | 1.46E+02 | [M+H]+ | Alkaloids |
| 69 | N-Feruloyltyramine; Moupinamide* | C18H19NO4 | 3.14E+02 | [M+H]+ | Alkaloids |
| 70 | N-Feruloylputrescine | C14H20N2O3 | 2.65E+02 | [M+H]+ | Alkaloids |
| 71 | N-Feruloyloctopamine | C18H19NO5 | 3.30E+02 | [M+H]+ | Alkaloids |
| 72 | N-Feruloyl-Cadaverine | C15H22N2O3 | 2.79E+02 | [M+H]+ | Alkaloids |
| 73 | N-Acetylputrescine | C6H14N2O | 1.31E+02 | [M+H]+ | Alkaloids |
| 74 | N-Acetylcadaverine | C7H16N2O | 1.45E+02 | [M+H]+ | Alkaloids |
| 75 | N-Acetyl-5-hydroxytryptamine | C12H14N2O2 | 2.19E+02 | [M+H]+ | Alkaloids |
| 76 | N-(4-oxopentyl)-acetamide | C7H13NO2 | 1.44E+02 | [M+H]+ | Alkaloids |
| 77 | N-(2-Hydroxy-4-methoxyphenyl)acetamide | C9H11NO3 | 1.82E+02 | [M+H]+ | Alkaloids |
| 78 | Myricetin-3,7,3'-trimethyl ether | C18H16O8 | 3.59E+02 | [M-H]- | Flavonoids |
| 79 | Myricetin | C15H10O8 | 3.19E+02 | [M+H]+ | Flavonoids |
| 80 | Methyleugenol | C11H14O2 | 1.79E+02 | [M+H]+ | Phenolic acids |
| 81 | Methyl gallate | C8H8O5 | 1.83E+02 | [M-H]- | Phenolic acids |
| 82 | Methyl dioxindole-3-acetate | C11H11NO4 | 2.22E+02 | [M+H]+ | Alkaloids |
| 83 | Methyl Syringate | C10H12O5 | 2.11E+02 | [M-H]- | Phenolic acids |
| 84 | Methyl L-pyroglutamate | C6H9NO3 | 1.44E+02 | [M+H]+ | Alkaloids |
| 85 | Methyl Cinnamate | C10H10O2 | 1.63E+02 | [M+H]+ | Phenolic acids |
| 86 | Methyl 4-hydroxybenzoate | C8H8O3 | 1.51E+02 | [M-H]- | Phenolic acids |
| 87 | Methyl 2,4-dihydroxyphenylacetate* | C9H10O4 | 1.81E+02 | [M-H]- | Phenolic acids |
| 88 | Matairesinol | C20H22O6 | 3.59E+02 | [M+H]+ | Lignans and Coumarins |
| 89 | Maltol | C6H6O3 | 1.27E+02 | [M+H]+ | Others |
| 90 | Lumichrome | C12H10N4O2 | 2.43E+02 | [M+H]+ | Alkaloids |
| 91 | Lirioresinol A | C22H26O8 | 4.17E+02 | [M-H]- | Lignans and Coumarins |
| 92 | Levopimaric acid* | C20H30O2 | 3.01E+02 | [M-H]- | Terpenoids |
| 93 | L-Tyramine | C8H11NO | 1.38E+02 | [M+H]+ | Alkaloids |
| 94 | L-Carnitine | C7H15NO3 | 1.62E+02 | [M+H]+ | Alkaloids |
| 95 | Kaurenoic Acid* | C20H30O2 | 3.01E+02 | [M-H]- | Terpenoids |
| 96 | Kaempferol (3,5,7,4'-Tetrahydroxyflavone) | C15H10O6 | 2.87E+02 | [M+H]+ | Flavonoids |
| 97 | Isovanillin | C8H8O3 | 1.53E+02 | [M+H]+ | Others |
| 98 | Isorhamnetin; 3'-Methoxy-3,4',5,7-Tetrahydroxyflavone | C16H12O7 | 3.15E+02 | [M-H]- | Flavonoids |
| 99 | Isopimaric acid* | C20H30O2 | 3.01E+02 | [M-H]- | Terpenoids |
| 100 | Isololiolide | C11H16O3 | 1.97E+02 | [M+H]+ | Terpenoids |
| 101 | Isolariciresinol | C20H24O6 | 3.59E+02 | [M-H]- | Lignans and Coumarins |
| 102 | Isohydroxymatairesinol | C20H22O7 | 3.73E+02 | [M-H]- | Lignans and Coumarins |
| 103 | Isofraxidin | C11H10O5 | 2.23E+02 | [M+H]+ | Lignans and Coumarins |
| 104 | Isoferulic Acid* | C10H10O4 | 1.93E+02 | [M-H]- | Phenolic acids |
| 105 | Isochlorogenic acid C | C25H24O12 | 5.17E+02 | [M+H]+ | Phenolic acids |
| 106 | Isochlorogenic acid B | C25H24O12 | 5.15E+02 | [M-H]- | Phenolic acids |
| 107 | Isochlorogenic acid A | C25H24O12 | 5.15E+02 | [M-H]- | Phenolic acids |
| 108 | Isoacteoside | C29H36O15 | 6.25E+02 | [M+H]+ | Phenolic acids |
| 109 | Indole-5-carboxylic acid* | C9H7NO2 | 1.60E+02 | [M-H]- | Alkaloids |
| 110 | Indole-3-carboxylic acid* | C9H7NO2 | 1.60E+02 | [M-H]- | Alkaloids |
| 111 | Indole-3-carboxaldehyde | C9H7NO | 1.46E+02 | [M+H]+ | Alkaloids |
| 112 | Indole-3-acetic acid (IAA) | C10H9NO2 | 1.76E+02 | [M+H]+ | Alkaloids |
| 113 | Indole | C8H7N | 1.18E+02 | [M+H]+ | Alkaloids |
| 114 | Imidazole-4-Acetic Acid* | C5H6N2O2 | 1.27E+02 | [M+H]+ | Alkaloids |
| 115 | Imidazol-1-yl-acetic acid* | C5H6N2O2 | 1.27E+02 | [M+H]+ | Alkaloids |
| 116 | Hydroxytyrosol | C8H10O3 | 1.53E+02 | [M-H]- | Phenolic acids |
| 117 | Hydroquinone | C6H6O2 | 1.09E+02 | [M-H]- | Phenolic acids |
| 118 | Hydrocinnamic acid | C9H10O2 | 1.49E+02 | [M-H]- | Phenolic acids |
| 119 | Homogentisic acid* | C8H8O4 | 1.67E+02 | [M-H]- | Phenolic acids |
| 120 | Homoeriodictyol | C16H14O6 | 3.03E+02 | [M+H]+ | Flavonoids |
| 121 | Histidinol | C6H11N3O | 1.42E+02 | [M+H]+ | Alkaloids |
| 122 | Histamine | C5H9N3 | 1.12E+02 | [M+H]+ | Alkaloids |
| 123 | Hexadecanamide | C16H33NO | 2.56E+02 | [M+H]+ | Alkaloids |
| 124 | Grandidentatin | C21H28O9 | 4.25E+02 | [M+H]+ | Phenolic acids |
| 125 | Geniposide | C17H24O10 | 3.89E+02 | [M+H]+ | Terpenoids |
| 126 | Gallocatechin | C15H14O7 | 3.07E+02 | [M+H]+ | Flavonoids |
| 127 | Gallic acid | C7H6O5 | 1.69E+02 | [M-H]- | Phenolic acids |
| 128 | Gallic Acid Ethyl Ester; Ethyl gallate | C9H10O5 | 1.97E+02 | [M-H]- | Phenolic acids |
| 129 | Fraxidin (8-Hydroxy-6,7-dimethoxycoumarin) | C11H10O5 | 2.23E+02 | [M+H]+ | Lignans and Coumarins |
| 130 | Fraxetin-8-O-glucoside (Fraxin) | C16H18O10 | 3.69E+02 | [M-H]- | Lignans and Coumarins |
| 131 | Fraxetin (7,8-Dihydroxy-6-methoxycoumarin) | C10H8O5 | 2.09E+02 | [M+H]+ | Lignans and Coumarins |
| 132 | Frambinone | C10H12O2 | 1.65E+02 | [M+H]+ | Others |
| 133 | Feruloylcholine | C15H22NO4+ | 2.80E+02 | [M]+ | Alkaloids |
| 134 | Ferulic acid* | C10H10O4 | 1.93E+02 | [M-H]- | Phenolic acids |
| 135 | Ferulic acid methyl ester | C11H12O4 | 2.07E+02 | [M-H]- | Phenolic acids |
| 136 | Eudesmic acid (3,4,5-trimethoxybenzoic acid) | C10H12O5 | 2.13E+02 | [M+H]+ | Phenolic acids |
| 137 | Eucommiol | C9H16O4 | 1.87E+02 | [M-H]- | Others |
| 138 | Ethylsalicylate | C9H10O3 | 1.67E+02 | [M+H]+ | Phenolic acids |
| 139 | Ethylparaben | C9H10O3 | 1.65E+02 | [M-H]- | Phenolic acids |
| 140 | Ethyl phenylacetate | C10H12O2 | 1.65E+02 | [M+H]+ | Phenolic acids |
| 141 | Ethyl ferulate | C12H14O4 | 2.21E+02 | [M-H]- | Phenolic acids |
| 142 | Ethyl caffeate | C11H12O4 | 2.07E+02 | [M-H]- | Phenolic acids |
| 143 | Esculetin (6,7-Dihydroxycoumarin) | C9H6O4 | 1.77E+02 | [M-H]- | Lignans and Coumarins |
| 144 | Eriodictyol (5,7,3',4'-Tetrahydroxyflavanone) | C15H12O6 | 2.89E+02 | [M+H]+ | Flavonoids |
| 145 | Epigallocatechin | C15H14O7 | 3.07E+02 | [M+H]+ | Flavonoids |
| 146 | Epicatechin | C15H14O6 | 2.91E+02 | [M+H]+ | Flavonoids |
| 147 | Emodin | C15H10O5 | 2.69E+02 | [M-H]- | Quinones |
| 148 | Embelin | C17H26O4 | 2.93E+02 | [M-H]- | Quinones |
| 149 | Dopamine | C8H11NO2 | 1.54E+02 | [M+H]+ | Alkaloids |
| 150 | Diosmetin (5,7,3'-Trihydroxy-4'-methoxyflavone)* | C16H12O6 | 3.01E+02 | [M+H]+ | Flavonoids |
| 151 | Dimethyl phthalate | C10H10O4 | 1.95E+02 | [M+H]+ | Phenolic acids |
| 152 | Diisooctyl Phthalate* | C24H38O4 | 3.91E+02 | [M+H]+ | Phenolic acids |
| 153 | Diisobutyl phthalate* | C16H22O4 | 2.79E+02 | [M+H]+ | Phenolic acids |
| 154 | Dihydrokaempferide | C16H14O6 | 3.03E+02 | [M+H]+ | Flavonoids |
| 155 | Dihydroferulic Acid | C10H12O4 | 1.95E+02 | [M-H]- | Phenolic acids |
| 156 | Dihydrocaffeic acid | C9H10O4 | 1.83E+02 | [M+H]+ | Phenolic acids |
| 157 | Dihydroactinidiolide | C11H16O2 | 1.81E+02 | [M+H]+ | Others |
| 158 | Digallic Acid | C14H10O9 | 3.21E+02 | [M-H]- | Phenolic acids |
| 159 | Decursinol | C14H14O4 | 2.47E+02 | [M+H]+ | Lignans and Coumarins |
| 160 | Daphnetin | C9H6O4 | 1.77E+02 | [M-H]- | Lignans and Coumarins |
| 161 | DL-2-Aminoadipic acid | C6H11NO4 | 1.62E+02 | [M+H]+ | Alkaloids |
| 162 | Coumarin-3-carboxylic Acid | C10H6O4 | 1.91E+02 | [M+H]+ | Lignans and Coumarins |
| 163 | Coumarin | C9H6O2 | 1.47E+02 | [M+H]+ | Lignans and Coumarins |
| 164 | Coniferin | C16H22O8 | 3.41E+02 | [M-H]- | Phenolic acids |
| 165 | Coniferaldehyde | C10H10O3 | 1.79E+02 | [M+H]+ | Others |
| 166 | Confertifoline | C15H22O2 | 2.33E+02 | [M-H]- | Terpenoids |
| 167 | Cinnamic acid | C9H8O2 | 1.47E+02 | [M-H]- | Phenolic acids |
| 168 | Cinnamamide | C9H9NO | 1.48E+02 | [M+H]+ | Alkaloids |
| 169 | Cimicifugamide | C25H31NO10 | 5.06E+02 | [M+H]+ | Alkaloids |
| 170 | Chrysosplenetin (5,4'-Dihydroxy-3,6,7,3'-tetramethoxyflavone)* | C19H18O8 | 3.75E+02 | [M+H]+ | Flavonoids |
| 171 | Chrysoeriol; 5,7,4'-Trihydroxy-3'-Methoxyflavone | C16H12O6 | 3.01E+02 | [M+H]+ | Flavonoids |
| 172 | Choline | C5H14NO+ | 1.04E+02 | [M]+ | Alkaloids |
| 173 | Catechin | C15H14O6 | 2.91E+02 | [M+H]+ | Flavonoids |
| 174 | Caffeoylcholine | C14H20NO4+ | 2.66E+02 | [M]+ | Alkaloids |
| 175 | Caffeic aldehyde | C9H8O3 | 1.65E+02 | [M+H]+ | Phenolic acids |
| 176 | Caffeic acid | C9H8O4 | 1.79E+02 | [M-H]- | Phenolic acids |
| 177 | Cadaverine | C5H14N2 | 1.03E+02 | [M+H]+ | Alkaloids |
| 178 | Butylphthalide* | C12H14O2 | 1.91E+02 | [M+H]+ | Others |
| 179 | Butyl isobutyl phthalate* | C16H22O4 | 2.79E+02 | [M+H]+ | Phenolic acids |
| 180 | Butin; 7,3',4'-Trihydroxyflavanone* | C15H12O5 | 2.73E+02 | [M+H]+ | Flavonoids |
| 181 | Betaine | C5H11NO2 | 1.18E+02 | [M+H]+ | Alkaloids |
| 182 | Benzylacetone | C10H12O | 1.49E+02 | [M+H]+ | Others |
| 183 | Benzoylmalic acid | C11H10O6 | 2.37E+02 | [M-H]- | Phenolic acids |
| 184 | Benzoic acid | C7H6O2 | 1.23E+02 | [M+H]+ | Phenolic acids |
| 185 | Benzamide | C7H7NO | 1.22E+02 | [M+H]+ | Alkaloids |
| 186 | Benzaldehyde | C7H6O | 1.07E+02 | [M+H]+ | Others |
| 187 | Apigenin; 4',5,7-Trihydroxyflavone | C15H10O5 | 2.71E+02 | [M+H]+ | Flavonoids |
| 188 | Apigenin-6-C-glucoside (Isovitexin)* | C21H20O10 | 4.33E+02 | [M+H]+ | Flavonoids |
| 189 | Antiarol; 3,4,5-Trimethoxyphenol | C9H12O4 | 1.85E+02 | [M+H]+ | Phenolic acids |
| 190 | Anthranilic Acid | C7H7NO2 | 1.38E+02 | [M+H]+ | Phenolic acids |
| 191 | Angelicin* | C11H6O3 | 1.87E+02 | [M+H]+ | Lignans and Coumarins |
| 192 | Androsin | C15H20O8 | 3.27E+02 | [M-H]- | Phenolic acids |
| 193 | Acetosyringone | C10H12O4 | 1.95E+02 | [M-H]- | Others |
| 194 | Acacetin-7-O-rutinoside (Linarin) | C28H32O14 | 5.93E+02 | [M+H]+ | Flavonoids |
| 195 | Acacetin | C16H12O5 | 2.85E+02 | [M+H]+ | Flavonoids |
| 196 | 9,19-Cyclolanost-24-en-3-ol (Cycloartenol) | C30H50O | 4.27E+02 | [M+H]+ | Terpenoids |
| 197 | 7-Methoxycoumarin | C10H8O3 | 1.77E+02 | [M+H]+ | Lignans and Coumarins |
| 198 | 7-Methoxy-5-Prenyloxycoumarin | C15H16O4 | 2.61E+02 | [M+H]+ | Lignans and Coumarins |
| 199 | 7-Hydroxycoumarin;Umbelliferone | C9H6O3 | 1.61E+02 | [M-H]- | Lignans and Coumarins |
| 200 | 7-Hydroxy-4-chromone | C9H6O3 | 1.63E+02 | [M+H]+ | Others |
| 201 | 7,8-Dihydroxy-4-phenylcoumarin | C15H10O4 | 2.55E+02 | [M+H]+ | Lignans and Coumarins |
| 202 | 7,8-Dihydroxy-4-methylcoumarin | C10H8O4 | 1.93E+02 | [M+H]+ | Lignans and Coumarins |
| 203 | 6-hydroxycoumarin | C9H6O3 | 1.63E+02 | [M+H]+ | Lignans and Coumarins |
| 204 | 6-Methylflavone | C16H12O2 | 2.37E+02 | [M+H]+ | Flavonoids |
| 205 | 6-MethylCoumarin | C10H8O2 | 1.61E+02 | [M+H]+ | Lignans and Coumarins |
| 206 | 6-Hydroxynicotinic acid | C6H5NO3 | 1.40E+02 | [M+H]+ | Alkaloids |
| 207 | 6-Hydroxy-7-methoxycoumarin* | C10H8O4 | 1.93E+02 | [M+H]+ | Lignans and Coumarins |
| 208 | 6-Hydroxy-4-methylcoumarin | C10H8O3 | 1.75E+02 | [M-H]- | Lignans and Coumarins |
| 209 | 6-Hydroxy-2'-methoxyflavone | C16H12O4 | 2.67E+02 | [M-H]- | Flavonoids |
| 210 | 6-Deoxyfagomine | C6H13NO2 | 1.32E+02 | [M+H]+ | Alkaloids |
| 211 | 6,7-Dihydroxy-4-methylcoumarin | C10H8O4 | 1.93E+02 | [M+H]+ | Lignans and Coumarins |
| 212 | 5-hydroxymaltol | C6H6O4 | 1.43E+02 | [M+H]+ | Others |
| 213 | 5-O-p-Coumaroylquinic acid* | C16H18O8 | 3.37E+02 | [M-H]- | Phenolic acids |
| 214 | 5-Methoxysalicylic acid | C8H8O4 | 1.69E+02 | [M+H]+ | Phenolic acids |
| 215 | 5-Hydroxymethylfurfural* | C6H6O3 | 1.27E+02 | [M+H]+ | Others |
| 216 | 5-Hydroxy-3,7,3',4'-tetramethoxyflavone (Retusin) | C19H18O7 | 3.59E+02 | [M+H]+ | Flavonoids |
| 217 | 5-Hydroxy-2-pyrrolidinone | C4H7NO2 | 1.02E+02 | [M+H]+ | Alkaloids |
| 218 | 5-Acetylsalicylic acid | C9H8O4 | 1.81E+02 | [M+H]+ | Phenolic acids |
| 219 | 5,7-Dimethoxycoumarin (Limettin)(Citropten) | C11H10O4 | 2.07E+02 | [M+H]+ | Lignans and Coumarins |
| 220 | 5,7-Dihydroxy-6,3',4',5'-tetramethoxyflavone (Arteanoflavone)* | C19H18O8 | 3.75E+02 | [M+H]+ | Flavonoids |
| 221 | 5,7-Dihydroxy-4-methylcoumarin | C10H8O4 | 1.93E+02 | [M+H]+ | Lignans and Coumarins |
| 222 | 5,7-Dihydroxy-3',4',5'-trimethoxyflavone | C18H16O7 | 3.45E+02 | [M+H]+ | Flavonoids |
| 223 | 5,7,8,4'-Tetramethoxyflavone | C19H18O6 | 3.43E+02 | [M+H]+ | Flavonoids |
| 224 | 5,6,7,7a-tetrahydro-4,4,7a-trimethyl-2(4H)-benzofuranone | C11H16O2 | 1.81E+02 | [M+H]+ | Others |
| 225 | 5,6,7,4'-Tetramethoxyflavone | C19H18O6 | 3.43E+02 | [M+H]+ | Flavonoids |
| 226 | 5,6,7,4'-Tetramethoxyflavanone | C19H20O6 | 3.45E+02 | [M+H]+ | Flavonoids |
| 227 | 5,4'-Dihydroxy-7-methoxyflavanone (Sakuranetin) | C16H14O5 | 2.87E+02 | [M+H]+ | Flavonoids |
| 228 | 4-Nitrophenol | C6H5NO3 | 1.40E+02 | [M+H]+ | Phenolic acids |
| 229 | 4-Methylcatechol | C7H8O2 | 1.23E+02 | [M-H]- | Phenolic acids |
| 230 | 4-Methylbenzaldehyde* | C8H8O | 1.19E+02 | [M-H]- | Others |
| 231 | 4-Methyl-5-thiazoleethanol | C6H9NOS | 1.44E+02 | [M+H]+ | Others |
| 232 | 4-Methoxysalicylic Acid | C8H8O4 | 1.67E+02 | [M-H]- | Phenolic acids |
| 233 | 4-Methoxyphenylpropionic acid | C10H12O3 | 1.79E+02 | [M-H]- | Phenolic acids |
| 234 | 4-MethoxycinnaMaldehyde | C10H10O2 | 1.63E+02 | [M+H]+ | Others |
| 235 | 4-Methoxybenzaldehyde | C8H8O2 | 1.37E+02 | [M+H]+ | Others |
| 236 | 4-Ketopinoresinol | C20H20O7 | 3.71E+02 | [M-H]- | Lignans and Coumarins |
| 237 | 4-Hydroxyquinoline | C9H7NO | 1.44E+02 | [M-H]- | Alkaloids |
| 238 | 4-Hydroxypyridine | C5H5NO | 9.60E+01 | [M+H]+ | Alkaloids |
| 239 | 4-Hydroxyphenyllactic Acid* | C9H10O4 | 1.81E+02 | [M-H]- | Phenolic acids |
| 240 | 4-Hydroxycoumarin | C9H6O3 | 1.61E+02 | [M-H]- | Lignans and Coumarins |
| 241 | 4-Hydroxybenzyl Alcohol | C7H8O2 | 1.23E+02 | [M-H]- | Phenolic acids |
| 242 | 4-Hydroxybenzoic acid* | C7H6O3 | 1.37E+02 | [M-H]- | Phenolic acids |
| 243 | 4-Hydroxybenzaldehyde | C7H6O2 | 1.21E+02 | [M-H]- | Others |
| 244 | 4-Hydroxyacetophenone | C8H8O2 | 1.35E+02 | [M-H]- | Others |
| 245 | 4-Hydroxy-3,5-diisopropylbenzaldehyde | C13H18O2 | 2.07E+02 | [M+H]+ | Phenolic acids |
| 246 | 4-Aminosalicylic acid | C7H7NO3 | 1.52E+02 | [M-H]- | Phenolic acids |
| 247 | 4-Aminobenzoic acid | C7H7NO2 | 1.38E+02 | [M+H]+ | Phenolic acids |
| 248 | 4-Allylcatechol | C9H10O2 | 1.49E+02 | [M-H]- | Phenolic acids |
| 249 | 4'-Hydroxypropiophenone | C9H10O2 | 1.49E+02 | [M-H]- | Others |
| 250 | 4'-Hydroxy-5,7-dimethoxyflavanone | C17H16O5 | 2.99E+02 | [M-H]- | Flavonoids |
| 251 | 3-hydroxyphenylacetic acid* | C8H8O3 | 1.51E+02 | [M-H]- | Phenolic acids |
| 252 | 3-amino-2-naphthoic acid* | C11H9NO2 | 1.88E+02 | [M+H]+ | Alkaloids |
| 253 | 3-[(1-Carboxyvinyl)oxy]benzoic acid | C10H8O5 | 2.07E+02 | [M-H]- | Phenolic acids |
| 254 | 3-O-p-Coumaroylquinic acid* | C16H18O8 | 3.37E+02 | [M-H]- | Phenolic acids |
| 255 | 3-O-Methylquercetin | C16H12O7 | 3.15E+02 | [M-H]- | Flavonoids |
| 256 | 3-O-Methylgallic acid | C8H8O5 | 1.83E+02 | [M-H]- | Phenolic acids |
| 257 | 3-O-Acetylpinobanksin | C17H14O6 | 3.13E+02 | [M-H]- | Flavonoids |
| 258 | 3-Methylsalicylic Acid | C8H8O3 | 1.51E+02 | [M-H]- | Phenolic acids |
| 259 | 3-Methylbenzaldehyde* | C8H8O | 1.19E+02 | [M-H]- | Others |
| 260 | 3-Methoxybenzoic acid | C8H8O3 | 1.51E+02 | [M-H]- | Phenolic acids |
| 261 | 3-Indolepropionic acid | C11H11NO2 | 1.90E+02 | [M+H]+ | Alkaloids |
| 262 | 3-Indoleacrylic acid* | C11H9NO2 | 1.88E+02 | [M+H]+ | Alkaloids |
| 263 | 3-Hydroxypyridine | C5H5NO | 9.60E+01 | [M+H]+ | Alkaloids |
| 264 | 3-Hydroxyphloretin | C15H14O6 | 2.91E+02 | [M+H]+ | Flavonoids |
| 265 | 3-Hydroxylup-20(29)-en-28-oic acid (Betulinic acid)* | C30H48O3 | 4.55E+02 | [M-H]- | Terpenoids |
| 266 | 3-Hydroxycinnamic Acid* | C9H8O3 | 1.63E+02 | [M-H]- | Phenolic acids |
| 267 | 3-Hydroxyanthranilic acid | C7H7NO3 | 1.54E+02 | [M+H]+ | Alkaloids |
| 268 | 3-Hydroxy-4-methoxybenzoic acid; Isovanillic Acid | C8H8O4 | 1.69E+02 | [M+H]+ | Phenolic acids |
| 269 | 3-Hydroxy-3-acetonyloxindole* | C11H11NO3 | 2.06E+02 | [M+H]+ | Alkaloids |
| 270 | 3-Chloroaniline | C6H6ClN | 1.28E+02 | [M+H]+ | Alkaloids |
| 271 | 3-Aminosalicylic acid | C7H7NO3 | 1.52E+02 | [M-H]- | Phenolic acids |
| 272 | 3-(4-Hydroxyphenyl)-propionic acid | C9H10O3 | 1.65E+02 | [M-H]- | Phenolic acids |
| 273 | 3-(3-Hydroxyphenyl)-propionic acid | C9H10O3 | 1.65E+02 | [M-H]- | Phenolic acids |
| 274 | 3,7-dihydroxy-4'-methoxyflavone | C16H12O5 | 2.85E+02 | [M+H]+ | Flavonoids |
| 275 | 3,7-Dihydroxychromen-4-one | C9H6O4 | 1.79E+02 | [M+H]+ | Others |
| 276 | 3,7-Di-O-methylquercetin | C17H14O7 | 3.29E+02 | [M-H]- | Flavonoids |
| 277 | 3,5-Dicaffeoylquinic acid | C25H24O12 | 5.15E+02 | [M-H]- | Phenolic acids |
| 278 | 3,5,7-Trihydroxyflavanone (Pinobanksin) | C15H12O5 | 2.71E+02 | [M-H]- | Flavonoids |
| 279 | 3,5,6,7,8,3',4'-Heptamethoxyflavone | C22H24O9 | 4.33E+02 | [M+H]+ | Flavonoids |
| 280 | 3,4-Dimethoxyphenyl acetic acid | C10H12O4 | 1.95E+02 | [M-H]- | Phenolic acids |
| 281 | 3,4-Dimethoxyphenol | C8H10O3 | 1.55E+02 | [M+H]+ | Phenolic acids |
| 282 | 3,4-Dimethoxycinnamic acid | C11H12O4 | 2.07E+02 | [M-H]- | Phenolic acids |
| 283 | 3,4-Dihydroxybenzoic acid (Protocatechuic acid)* | C7H6O4 | 1.53E+02 | [M-H]- | Phenolic acids |
| 284 | 3,4-Dihydroxybenzoic Acid Ethyl Ester (Protocatechuic acid ethyl ester) | C9H10O4 | 1.81E+02 | [M-H]- | Phenolic acids |
| 285 | 3,4-Dihydroxybenzeneacetic acid* | C8H8O4 | 1.67E+02 | [M-H]- | Phenolic acids |
| 286 | 3,4-Dihydroxyacetophenone | C8H8O3 | 1.51E+02 | [M-H]- | Others |
| 287 | 3,4-Dihydrocoumarin | C9H8O2 | 1.47E+02 | [M-H]- | Lignans and Coumarins |
| 288 | 3,4,5-Trimethoxycinnamic acid | C12H14O5 | 2.37E+02 | [M-H]- | Phenolic acids |
| 289 | 3,4'-Dihydroxyflavone | C15H10O4 | 2.55E+02 | [M+H]+ | Flavonoids |
| 290 | 3,3',4-O-Trimethylellagic acid | C17H12O8 | 3.45E+02 | [M+H]+ | Tannins |
| 291 | 3,25-Epoxy-3-hydroxyolean-18-en-28-oic acid (Semimoronic acid) | C30H46O4 | 4.69E+02 | [M-H]- | Terpenoids |
| 292 | 3,23-Dihydroxyolean-12-en-28-oic acid (Hederagenin)* | C30H48O4 | 4.71E+02 | [M-H]- | Terpenoids |
| 293 | 3,23-Dihydroxy-30-noroleana-12,20(29)-dien-28-oic acid (30-Norhederagenin) | C29H44O4 | 4.55E+02 | [M-H]- | Terpenoids |
| 294 | 3',7-dihydroxy-4'-methoxyflavone | C16H12O5 | 2.85E+02 | [M+H]+ | Flavonoids |
| 295 | 3',4',7-Trihydroxyflavone | C15H10O5 | 2.71E+02 | [M+H]+ | Flavonoids |
| 296 | 2-Piperidone | C5H9NO | 1.00E+02 | [M+H]+ | Alkaloids |
| 297 | 2-Picoline; 2-Methylpyridine | C6H7N | 9.41E+01 | [M+H]+ | Alkaloids |
| 298 | 2-Phenylethylamine | C8H11N | 1.22E+02 | [M+H]+ | Alkaloids |
| 299 | 2-Phenylethy-1-O-β-D-glucoside | C14H20O6 | 2.85E+02 | [M+H]+ | Phenolic acids |
| 300 | 2-Pentyl-3-phenyl-2-propenal | C14H18O | 2.03E+02 | [M+H]+ | Others |
| 301 | 2-Oxo-3,4-dihydro-1H-quinoline-3-carboxylic acid | C10H9NO3 | 1.92E+02 | [M+H]+ | Alkaloids |
| 302 | 2-Methoxy-4-methylphenol | C8H10O2 | 1.39E+02 | [M+H]+ | Phenolic acids |
| 303 | 2-Hydroxycinnamic acid* | C9H8O3 | 1.63E+02 | [M-H]- | Phenolic acids |
| 304 | 2-Hydroxybenzaldehyde (Salicylaldehyde) | C7H6O2 | 1.21E+02 | [M-H]- | Phenolic acids |
| 305 | 2-Hydroxy-3-phenylpropanoic acid | C9H10O3 | 1.65E+02 | [M-H]- | Phenolic acids |
| 306 | 2-Hydroxy-3-(4-Hydroxyphenyl)Propanoic Acid* | C9H10O4 | 1.81E+02 | [M-H]- | Phenolic acids |
| 307 | 2-Feruloyl-sn-glycerol | C13H16O6 | 2.69E+02 | [M+H]+ | Phenolic acids |
| 308 | 2-Ethyl-2,6,6-trimethylpiperidin-4-one | C10H19NO | 1.70E+02 | [M+H]+ | Alkaloids |
| 309 | 2-Decanol* | C10H22O | 1.57E+02 | [M-H]- | Others |
| 310 | 2-Benzoxazolinone | C7H5NO2 | 1.36E+02 | [M+H]+ | Alkaloids |
| 311 | 2-Aminophenol | C6H7NO | 1.10E+02 | [M+H]+ | Alkaloids |
| 312 | 2-Amino-3-methoxybenzoic acid | C8H9NO3 | 1.68E+02 | [M+H]+ | Phenolic acids |
| 313 | 2-(Formylamino)benzoic acid | C8H7NO3 | 1.64E+02 | [M-H]- | Phenolic acids |
| 314 | 2-(Acetylamino)-3-phenyl-2-propenoic acid* | C11H11NO3 | 2.06E+02 | [M+H]+ | Alkaloids |
| 315 | 2,6-Dimethoxybenzoic acid | C9H10O4 | 1.83E+02 | [M+H]+ | Phenolic acids |
| 316 | 2,6-Dimethoxybenzaldehyde | C9H10O3 | 1.65E+02 | [M-H]- | Others |
| 317 | 2,6-Di-tert-butylphenol* | C14H22O | 2.05E+02 | [M-H]- | Phenolic acids |
| 318 | 2,5-dihydroxy-1-methoxy-anthraquinone | C15H10O5 | 2.69E+02 | [M-H]- | Quinones |
| 319 | 2,5-Dihydroxybenzoic acid; Gentisic Acid* | C7H6O4 | 1.53E+02 | [M-H]- | Phenolic acids |
| 320 | 2,5-Dihydroxyacetophenone | C8H8O3 | 1.51E+02 | [M-H]- | Others |
| 321 | 2,4-Dinitrophenol | C6H4N2O5 | 1.83E+02 | [M-H]- | Phenolic acids |
| 322 | 2,4-Dihydroxybenzoic acid | C7H6O4 | 1.53E+02 | [M-H]- | Phenolic acids |
| 323 | 2,4-Dihydroxybenzaldehyde | C7H6O3 | 1.37E+02 | [M-H]- | Others |
| 324 | 2,4-Di-Tert-Butylphenol* | C14H22O | 2.05E+02 | [M-H]- | Phenolic acids |
| 325 | 2,4,6-Trihydroxybenzoic acid | C7H6O5 | 1.71E+02 | [M+H]+ | Phenolic acids |
| 326 | 2,4,6,6-Tetramethyl-3(6H)-pyridinone | C9H13NO | 1.52E+02 | [M+H]+ | Alkaloids |
| 327 | 2,3-Dihydroxyurs-12-en-29-oic acid (Maslinic acid)* | C30H48O4 | 4.71E+02 | [M-H]- | Terpenoids |
| 328 | 2,3-Dihydroxyurs-12-en-28-oic acid (Corosolic acid)* | C30H48O4 | 4.71E+02 | [M-H]- | Terpenoids |
| 329 | 2,3-Dihydroxylup-20(29)-en-28-oic acid (Alphitolic acid)* | C30H48O4 | 4.71E+02 | [M-H]- | Terpenoids |
| 330 | 2,3-Dihydroxybenzoic Acid* | C7H6O4 | 1.53E+02 | [M-H]- | Phenolic acids |
| 331 | 2,3,4-Trihydroxybenzoic acid | C7H6O5 | 1.69E+02 | [M-H]- | Phenolic acids |
| 332 | 2,3,19-Trihydroxyurs-12-en-28-oic acid (Tormentic acid) | C30H48O5 | 4.87E+02 | [M-H]- | Terpenoids |
| 333 | 2,19-Dihydroxy-3-oxours-12-en-28-oic acid | C30H46O5 | 4.85E+02 | [M-H]- | Terpenoids |
| 334 | 2',6'-Dihydroxyacetophenone | C8H8O3 | 1.53E+02 | [M+H]+ | Phenolic acids |
| 335 | 2',4'-Dimethylacetophenone | C10H12O | 1.49E+02 | [M+H]+ | Others |
| 336 | 2',4'-Dihydroxyacetophenone | C8H8O3 | 1.51E+02 | [M-H]- | Others |
| 337 | 2',4',6'-Trihydroxyacetophenone | C8H8O4 | 1.67E+02 | [M-H]- | Phenolic acids |
| 338 | 16,23:16,30-Diepoxydammar-24-ene-3,20-diol (Jujubogenin)* | C30H48O4 | 4.71E+02 | [M-H]- | Terpenoids |
| 339 | 1-O-Cinnamoyl-β-D-glucose | C15H18O7 | 3.09E+02 | [M-H]- | Phenolic acids |
| 340 | 1-Methylhistamine | C6H11N3 | 1.26E+02 | [M+H]+ | Alkaloids |
| 341 | 1-Methyl-6-Oxo-1,6-Dihydropyridine-3-Carboxamide | C7H8N2O2 | 1.53E+02 | [M+H]+ | Alkaloids |
| 342 | 1-Hydroxyanthraquinone | C14H8O3 | 2.25E+02 | [M+H]+ | Quinones |
| 343 | 1-Feruloyl-sn-glycerol | C13H16O6 | 2.69E+02 | [M+H]+ | Phenolic acids |
| 344 | 1-Decanol* | C10H22O | 1.57E+02 | [M-H]- | Others |
| 345 | 1-(4-Methoxyphenyl)-1-propanol | C10H14O2 | 1.65E+02 | [M-H]- | Phenolic acids |
| 346 | 1,4-Dihydro-1-Methyl-4-oxo-3-pyridinecarboxamide | C7H8N2O2 | 1.51E+02 | [M-H]- | Alkaloids |
| 347 | 1,3-O-Diferuloylglycerol* | C23H24O9 | 4.45E+02 | [M+H]+ | Phenolic acids |
| 348 | 1,3-O-Di-p-Coumaroylglycerol | C21H20O7 | 3.83E+02 | [M-H]- | Phenolic acids |
| 349 | 1,2-O-Diferuloylglycerol* | C23H24O9 | 4.45E+02 | [M+H]+ | Phenolic acids |

**Supplementary Table 6**

**Core genes in PPI network ranked by Degree method**

| **Rank** | **Degree** | **name** |
| --- | --- | --- |
| 1 | 57 | CCL2 |
| 2 | 51 | PTPRC |
| 3 | 49 | EGF |
| 4 | 42 | IGF1 |
| 5 | 33 | SPP1 |
| 6 | 32 | CD36 |
| 7 | 32 | VWF |
| 8 | 30 | COL1A1 |
| 9 | 30 | CCR2 |
| 10 | 28 | CD40LG |
| 11 | 27 | CSF1R |
| 12 | 26 | JAK2 |
| 13 | 25 | CCR5 |
| 14 | 24 | CXCR2 |
| 15 | 24 | MMP1 |
